# Analyzing the Prevalence of and Factors Associated with Road Traffic Crashes (RTCs) among Motorcyclists in Bangladesh

**DOI:** 10.1155/2024/7090576

**Published:** 2024-05-09

**Authors:** Md. Mamun Miah, Biton Chakma, Kabir Hossain

**Affiliations:** Department of Statistics, Noakhali Science and Technology University, Noakhali 3814, Bangladesh

## Abstract

**Methods:**

A cross-sectional survey was conducted using a structured questionnaire involving 402 motorcyclists from four major southeastern towns, comprising 350 (86.07%) males and 52 (12.93%) females. The chi-square test was applied in bivariate analysis, and binary multivariable logistic regression was performed to determine the risk factors of road traffic crashes.

**Results:**

This study's findings revealed that the overall reported prevalence of road traffic crashes involving motorcycle drivers over one year was 68.66%. Multivariable logistic regression analysis revealed several factors that significantly impacted road traffic crashes. These factors included driving without a valid driving license, the young age (<20) of motorcyclists, driving in rainy weather, exceeding the speed limit, per-week working hours, smoking status, motorcycle ownership, the brand of motorcycle, and not wearing a helmet while driving.

**Conclusion:**

The study findings highlight the need for improving motorcycle safety by implementing measures such as imposing per-week work hour limits for riders, enforcing traffic regulations, and promoting helmet use among motorcycle drivers. The results of this study draw attention to the Bangladesh Road Transport Authority (BRTA) and motorcycle drivers in the country to decrease motorcycle crashes and the severity of injuries by implementing efficient guidelines and strategies for driving motorcycles. The findings of this study can assist policymakers and concerned authorities in taking the essential steps to lessen road traffic crashes among motorcyclists in Bangladesh.

## 1. Introduction

Motorcycles are a standard mode of conveyance worldwide, with millions of people using them for daily commutes, goods and public transit, relaxation, and sporting activities. According to the International Transport Forum, over 440 million motorcycles were used worldwide in 2019 [1]. One of the primary reasons for motorcycles' popularity is their low cost, fuel savings, speed, and agility in busy cities, and that is why in many developing countries, like Bangladesh, motorcycles are a cheaper way to get around than cars [2]. According to a report by the International Transport Forum (ITF) in 2019, motorcycles and scooters made up approximately 20% of motor-driven two-wheelers worldwide. They were the primary mode of motorized transport in many low- and middle-income countries. The most worrying statistic in 2023 is that Bangladesh has the highest proportion of motorcycle fatalities, 28 per 10,000 motorcycles [3].

The World Health Organization (WHO) stated that approximately 1.3 million individuals die annually as a consequence of traffic accidents. In contrast, pedestrians, bicyclists, and motorcyclists were involved in nearly 50% of all road traffic fatalities and 43% in Europe in 2018 [4]. According to the European Road Safety Observatory study from 2020, motorcycles are responsible for almost 19% of all traffic fatalities in the United Kingdom, compared to a lower rate of 15.5% across the European Union [5]. In Australia, population-based rates of motorcycle crash injuries increased at an average rate of 9.6% annually [6]. Most of the deaths and severe injuries on China's roads are caused by motorbike accidents [7, 8]. As compared to other Asian nations, Bangladesh has one of the highest average road traffic accidents (RTAs) fatality rates in 2023 [9]. RTAs are rising due to the unexpectedly significant increase in the number of motor vehicles and the growth of cities and towns [10]. In many developing regions, there are fewer opportunities for receiving extensive training in driving and road safety due to the rising number of motorcycle owners, which causes RTAs and increases traffic [11]. Motorcycle-related problems are becoming more frequent in Asian countries, and RTAs related to motorcycles account for more than half of all Asian traffic accidents [12].

However, numerous studies have been conducted to investigate the risk factors such as drinking alcohol and excess speed while driving, the number of individuals involved, the lighting conditions, and the presence of a motorcycle and a pedestrian crossing associated with motorcycle drivers in developed and developing nations. [13]. Shockingly, 84% (16 out of 19) of prehospital deaths were due to the absence of helmet use [14]. The major contributory factors in severe and fatal road traffic crashes involving motorcycles are road type and weather [15]. Liu et al. examined four higher-quality studies and found that helmets were predicted to decrease the probability of death by 42% and the risk of head injuries by 69% [16]. Approximately 63.4% of the students reported having a ridden experience before turning 18. Additional research is necessary to evaluate the effectiveness of different road safety interventions in reducing motorcycle accidents and injuries, especially among teenagers.

Worldwide, biking is considered a sport and another part of a regular daily activity. Both male and female riders ride the bike for their daily necessity, commuting, touring, or sporting. But in Bangladesh, very few female motorcyclists are available here. Females and males have fundamental differences in preferences and behaviours [17]. For example, females tend to be more risk-averse, less prone to competitive environments, and respond to financial incentives differently from males. During COVID-19, most of the countries that were highly affected were in lockdown. Lockdown disrupts supply chains and logistics in rural areas, negatively impacting rural households' financial welfare [18]. The pandemic had a significant impact on the Chinese economy and the economies of the countries that were heavily affected. To limit the adverse effects of the pandemic, governments all around the world have adopted a range of nonpharmaceutical interventions. In the early stages of the COVID-19 pandemic, Chinese central and local governments enacted lockdowns in most parts of China to limit the spread of the virus. In China, FinTech lending provides accessible and personalized credit services to people who cannot satisfy their credit demands from banks [19]. Share business has also lost its popularity during COVID-19. The connectivity of stock markets reflects the information efficiency of capital markets and contributes to interior risk contagion and spillover effects [20]. During or after the pandemic, motorcycles have become one of the neediest transportation mediums in Bangladesh.

Our paper is connected to a growing literature investigating the factors linked with road traffic crashes [21–29]. According to a Nigerian study of 224 commercial motorcyclists, 46% have been involved in at least one traffic accident. Poor road conditions and refusal to obey traffic signs were discovered to be two key factors contributing to frequent collisions [30]. In another investigation, Agyekum-Boamah [31] used questionnaires to collect information from 200 motorcycle riders in Ghana's Greater Accra, Volta, and Upper East regions. According to the findings, around 50% of motorcyclists had collisions with other vehicles on the road, and about 80% said they had been involved in several accidents. More recently, Wankie et al. conducted cross-sectional research among consenting commercial motorbike riders in Bamenda, Cameroon [29]. The study sought to determine the proportion of self-reported crashes and investigate the factors that contribute to such accidents. Out of 552 participants, 77.4% said they had been in a crash as commercial motorcycle riders. In addition, 21.5% of self-reported crashes occurred within the previous 12 months. In Maoming, South China, Wu and Loo [32] investigated the characteristics of motorcycle taxis and nonprofessional riders. This study also examined attitudes about road traffic safety and self-reported riding behaviours in these two groups. They discovered that motorcycle taxi drivers were more likely to encounter risks to road safety than nonprofessional motorcyclists.

The investigation of motorcyclists' potential safety risks needs to be more detailed. Previous studies have mainly concentrated on major cities or metropolitan areas like Dhaka in Bangladesh [33] and Ha Noi, Da Nang, and Ho Chi Minh in Vietnam [34]. To the best of our knowledge, this work represents the first study to analyze the prevalence and determinants of road traffic crashes among motorcyclists in both towns and municipal areas of Bangladesh. The main objective of our research is to investigate the prevalence of road traffic crashes and identify the factors associated with road traffic crashes among motorcyclists in Bangladesh. From these perspectives, the effectiveness of existing road safety interventions, such as helmet laws, speed limits, and road infrastructure improvements, is badly needed to reduce motorcycle-related road traffic crashes and injuries. This study helps raise public knowledge regarding the significance of safe driving practices and the dangers of certain behaviours in reducing road traffic crashes caused by motorcycles.

This paper is structured as follows. The next section provides a methodology for the research, including the study site, sample size, and sampling technique. Data analysis is followed by results, discussion, and conclusion. A set of results is then presented, comprising descriptive statistics, a chi-square test, and logistic regression model results.

## 2. Methodology

### 2.1. Study Site

**Figure 1:**
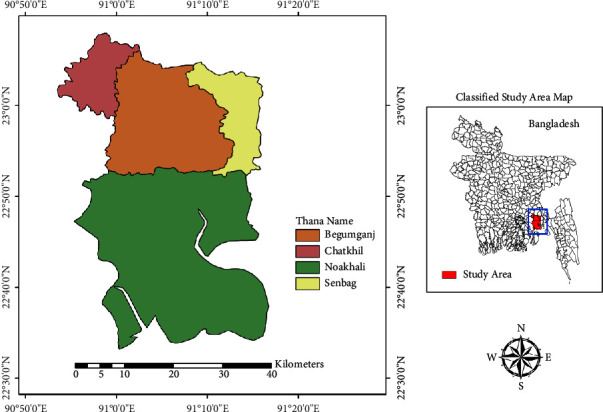
Study site of the study.

Bangladesh, the eighth-most populous country in the world, is located on the Indian subcontinent in south-central Asia. It has eight administrative divisions and sixty-four districts. Road traffic fatalities and injuries within Bangladesh have emerged as a muted epidemic. These vehicular accidents not only result in personal tragedies for families but also act as obstacles to economic growth and the promotion of sustainable development. Annually, road collisions claim the lives of over 4,000 Bangladeshi citizens while leaving an undetermined number with severe incapacitations. As reported by the Bangladesh Road Transport Authority (BRTA), the number of registered motorcycles in Bangladesh is 3,897,031. Information from the Bangladesh University of Engineering and Technology (BUET) Accident Research Institute (ARI) indicates that motorcycles constitute 62% of the overall vehicle count on the country's roads, with approximately 26 accidents occurring for every 10,000 motorcycles. Four Upazilas (towns) were chosen for sampling due to their many motorbikes, busy junctions, and motorcycle parking areas. These are Noakhali Sadar (22°50′N, 91°6′E), Begumganj (22.95°N, 91.10°E), Chatkhil (23.0500°N, 90.9583°E), and Senbagh (22°59′N, 91°14′E). The total area, total population, and officially registered motorcycles in Noakhali Sadar, Begumganj, Chatkhil, and Senbagh are 336.06 km^2^, 6,84,842, 60,514; 426.05 km^2^, 5,67,903, 49,785; 133.89 km^2^, 4,86,904, 41,234; and 159.36 km^2^, 3,87,393, 29,367, respectively. The study area has been shown in Figure 1.

### 2.2. Sample Size and Sampling Technique

The present study collected data using a convenience sampling approach from 10 March 2022 through 30 April 2022 through field and online surveys. The process of gathering samples by selecting conveniently placed samples throughout a site is known as convenience sampling. Respondents who are “convenient” for the researcher are used in this situation. There is no pattern in how these respondents are found; they may be seen by asking individuals who appear on the street, in a public place, or at the workplace. The Noakhali branch of the Bangladesh Road Transport Authority (BRTA) reported that there was a total of 180,900 motorcycles that were officially registered in the four towns of Noakhali. The sample size for this study was calculated using Slovin's formula, *n* = *N*/(1 + Ne2), with a confidence interval of 95% and a margin of error of 5% considered. As a result, it was determined that a sample size of 400 was required. Data were collected using a structured Google questionnaire in the field survey. Before commencing the field survey, comprehensive information describing the objectives of the study and the survey was provided to each surveyor.

Moreover, extensive guidelines were provided regarding the appropriate methodology to conduct the survey effectively. The field surveyors approached 510 motorcyclists, and 245 responded, indicating a response rate of 48%. 131, 35, 46, and 33 samples were collected from Noakhali Sadar, Begumganj, Chatkhil, and Senbagh. While the field survey was underway, a Google questionnaire was uploaded to the four separate Facebook groups (Praner Sohor Maijdee, Alokito Begumganj, Amader Chatkhil, and Amader Senbagh) in the four towns. The names of Facebook groups already exist the different authorities in Bengali based on their community demands whose meanings in English are “Maijdee is the city of life,” “Illuminated Begumganj,” “Our Chatkhil,” and “Our Senbagh.” The online survey showed a response rate of 42.61%, with 176 out of 413 motorcyclists sharing their opinions.

The questionnaire used in the study was divided into four sections. The first section aimed to collect demographic information about the motorcyclists, such as their age, sex, residence, marital status, education, occupation, years of driving experience, motorbike ownership, and motorcycle licenses. In the questionnaire, the age variable was kept categorical and measured in years. The second section consisted of closed-ended questions about respondents' work schedules and commuting times. The third section polled bikers on their thoughts and routines concerning safety on the road, including infractions such as failing to use a turn signal, swerving in and out of traffic, going too fast, passing a slower vehicle without checking for oncoming traffic, talking on the phone, drinking alcohol, smoking cigarettes, carrying an excessive number of passengers, not wearing a helmet, and running a red light. The last section asked motorcyclists about their experiences in road traffic crashes throughout the previous year.

From 10 March 2022 to 30 April 2022, 421 motorcyclists' data out of a sampling frame of approximately 180,900 registered motorcycles were gathered, comprising 245 in field-based survey respondents and 176 online survey respondents. Due to the availability of an electronic version of the Google questionnaire and face-to-face interviews with respondents, the field survey results contained all the data. However, 19 responses to the online survey lacked meaningful value, and in some cases, critical variables needed to be included. After eliminating these instances of missing data, 402 valid responses remained for analysis, of which 350 (86.07%) were men and 52 (12.93%) were women.

### 2.3. Data Analysis

Before being imported into SPSS for analysis, the obtained data were coded in a Microsoft Excel spreadsheet and checked for completeness and accuracy. Any observations with missing values were excluded from the study. The prevalence of road traffic crashes was evaluated using descriptive statistics with 95% confidence intervals and categorizing the results by variables such as age and gender. We employed a binary logistic regression model due to the categorical structure of the response variable (“Have you faced any motorcycle traffic crashes over the last one-year period?”) with binary outcomes (either “no” or “yes”). The aim was to determine the influential factors linked to road traffic crashes. Logistic regression (LR), like linear regression, is a statistical approach. While a logistic regression seeks an equation for predicting the outcomes of a binary variable, Y, based on one or more predictor variables, *X*, it differs from linear regression in that the predictor variables can include categorical and continuous data. The log odds ratio is used instead of probabilities in LR, and the final model is fitted using an iterative maximum likelihood technique rather than least squares. The equation of a logistic regression model with *i*th predictors is *Logit* (*P*)=ln(*P*/1 − *P*)=*α*_0_+*β*_1_*X*_1_+*β*_2_*X*_2_+*β*_3_*X*_3_+…+*β*_*i*_*X*_*i*_, where logit is the natural logarithm of the odds, *P* is the probability of having the outcome, and *P*/1 − *P* is the odds of the outcome. The dependent variable *Y* represents the occurrence of crashes (*Y*=1) versus noncrashes (*Y*=0). The variable *P* denotes the probability of a crash occurring. The intercept is represented by the symbol *α*_0_, while *β*_*i*_ represents the coefficient value of the model. Lastly, *X*_*i*_ represents the independent variables used in the present study. The model that was developed displayed the coefficient (B), significance level (*p* value), and odds ratio (OR) of the independent variables.

The coefficient (B) value of a categorical independent variable indicates the presence of a positive or negative relationship with the dependent variable for a particular level compared to the reference level of that variable. The probability of accidents increases with a positive coefficient (B). The model's results can also be interpreted based on the odds ratio (OR) value. In this context, an odds ratio (OR) greater than one indicates that the probability of crashes increases by a factor of *X* for a specific independent variable relative to the reference level of that independent variable while keeping other variables constant. Similarly, suppose that the value of *X* is smaller than one. In that case, it indicates that the probability of crashes decreases by a factor of *X* for a specific independent variable relative to the reference level of that independent variable while keeping other variables constant. The statistical software IBM SPSS Statistics 25 was used to conduct the analysis.

## 3. Results

### 3.1. Prevalence of Crashes among Motorcycle Drivers

In bivariate analysis, the chi-square test was employed to find the association between road traffic crashes and their associated factors. The factors related to RTCs can be categorized into four groups: demographic, working history, lifestyle/behavioural, and miscellaneous factors. Demographic factors include age (*χ*^2^=10.427, *p* *value* < 0.05), marital status (*χ*^2^=15.836, *p* *value* < 0.001), occupation (*χ*^2^=13.804, *p* *value* < 0.001), and education level (*χ*^2^=11.247, *p* *value* < 0.05), and all of them were found to have a significant association with RTCs (Table 1). Working history factors include employment status (*χ*^2^=13.233, *p* *value* < 0.001), driving experience (*χ*^2^=30.294, *p* *value* < 0.001), motorcycle ownership (*χ*^2^=36.7, *p* *value* < 0.001), daily travel distance (*χ*^2^=62.034, *p* *value* < 0.001), and weekly working hours (*χ*^2^=61.33, *p* *value* < 0.001), which also showed a significant association with RTCs (Table 1). Lifestyle and behavioral factors that were associated with road traffic crashes were talking on the phone while riding (*χ*^2^=32.944, *p* *value* < 0.001), smoking status (*χ*^2^=7.888, *p* *value* < 0.05), and smoking while driving (*χ*^2^=45.076, *p* *value* < 0.001) (Table 1). There were five miscellaneous associated factors among nine with RTCs: helmet use (*χ*^2^=63.519, *p* *value* < 0.001), motorcycle brand (*χ*^2^=31.334, *p* *value* < 0.001), driving license (*χ*^2^=50.856, *p* *value* < 0.001), exceeding speed limit (*χ*^2^=39.193, *p* *value* < 0.001), and red light running (*χ*^2^=9.034, *p* *value* < 0.05) (Table 1). All these factors were found to have a substantial association with RTCs after extensive research.

Most of the 402 legitimate survey respondents were men (350 out of 402, or 87.06%). Almost half of the motorcyclists, 180 (44.78%), were between the ages of 20 and 29. Most of the respondents (357, or 88.81%) were not students (45, or 11.19%), and most of them had part-time jobs (66%) since most of them used motorcycles for personal and career purposes. Most bikers (68.41%) bought their bikes with their own money instead of getting them as gifts from their family. Descriptive statistics for the sample, including information on the prevalence of crashes and noncrashes among motorcyclists, are displayed in Table 1.

The proportion of surveyed motorcycle drivers who reported having experienced road traffic crashes was 68.66%. RTCs were more likely to happen to drivers who lived in cities, with a rate of 74.25%, than to drivers who lived in rural areas, with a rate of 60.95%. Notably, motorcyclists that were students reported a significantly lower incidence of crashes, i.e., 44.44%, compared to other drivers, i.e., 71.71%. Additionally, drivers with an educational qualification above high school reported a lower prevalence of crashes. Specifically, those with an educational qualification below high school reported the lowest incidence of crashes, with only 44.74% having a crash in the past year. The incidence of crashes was observed to be considerably higher due to an increase in the average distance travelled each day. The drivers who covered 30 to 50 kilometres per day had the highest crash rate at 84.69%, followed by those who travelled 20 to 30 kilometres per day at 58.09%, more than 50 kilometres per day at 36.84%, and less than 20 kilometres per day at 14%. The data did not reveal a corresponding trend for the number of hours worked per week among the motorcyclists. Although drivers who worked 40 to 50 hours per week had the highest rate of 80.07%, followed by those who worked 50 to 60 hours per week at 45.28%, the prevalence of RTCs reported by motorcyclists who worked less than 30 hours and more than 60 hours per week was 40.91% and 34.15%, respectively.

Regarding motorcyclists' lifestyle, those who smoke while driving had the highest incidence of crashes, i.e., 89.12%, compared to those who do not smoke while driving, i.e., 56.86%. The prevalence of crashes was significantly higher among drivers who reported regularly using their mobile phones while driving, with a rate of 100%. In comparison, those who reported using their mobile phones often while driving had a crash prevalence rate of 86.67%, and those who reported sometimes using their phones while driving had a rate of 59.09%.

Of the miscellaneous factors, five were strongly linked to road traffic crashes, according to the survey. The prevalence rate of crashes was higher among those who drove a motorcycle without wearing a helmet (87.37%) than those who wore a helmet. The prevalence rate also differed according to the brand of motorcycle used, with Suzuki having the highest rate (85.82%), followed by other brands (64%), Honda (56.98%), and Yamaha (56.00%). Interestingly, the prevalence rates for Honda and Yamaha were almost equal (56%). Those with a motorcycle license had a higher prevalence rate (76.36%) than those without one (33.33%). The reason for this occurrence may be that, despite the Bangladesh Road Transport Authority (BRTA) stipulating that individuals must satisfy certain conditions, such as being 18 years or older and successfully passing both a written and practical driving test to acquire a motorcycle driving license, a significant number of people still acquire licenses with ease through illegitimate means involving brokers and bribes. Additionally, the prevalence rate was higher (91.96%) for drivers who exceeded the speed limit than those who did not (59.66%). Moreover, the prevalence rate was high (70.03%) for those who drove with a red light on compared to those who did not (33.33%).

### 3.2. Factors Influencing Road Traffic Crashes

Logistic regression analysis of the impacts of different variables related to motorcycle drivers on the likelihood of road traffic crashes is shown in Table 2. The study finds that nine factors significantly affect RTCs. These factors include the age of the driver, type of motorcycle ownership, weekly working hours, smoking status, helmet usage, brand of motorcycle, possession of a driving license, exceeding the speed limit, and prevailing weather conditions. According to the model, drivers aged 21 to 29 have the highest probability of being involved in road traffic crashes compared to those who are 50 or older. (*OR* : 0.118, *p* *value* < 0.05). The probability of motorcycle drivers being involved in traffic crashes is lower for those who inherit their motorcycles compared to those who purchase their own, as per the study's findings (*OR* : 0.415, *p* *value* < 0.05). The analysis shows that drivers who work for at least 40 hours and less than 50 hours per week increase the likelihood of being in a road traffic crash (*OR* : 4.113, *p* *value* < 0.05). However, those who work between 11 and 15 hours per week are less likely to be involved in RTCs than those who work more than 20 hours per week. Motorcycle drivers who smoke occasionally have a lower probability of experiencing severe RTCs compared to those who smoke regularly, according to the study's findings (*OR* : 0.284, *p* *value* < 0.05).

Helmet usage is found to be a crucial factor in preventing road traffic crashes, with drivers who do not use a helmet being three times more likely to be involved in a crash (*OR* : 3.423, *p* *value* < 0.05). Suzuki motorcycle users are almost 2 times more likely to be in RTCs than Yamaha, Honda, or other brand users (*OR* : 2.744, *p* *value* < 0.05). Having a driving license is an important factor in preventing traffic crashes, as those who do not possess a license are more likely to be involved in a road traffic crash (*OR* : 0.335, *p* *value* < 0.05). According to the analysis, drivers who did not exceed the speed limit were 0.27 times less likely to be involved in RTCs compared to those who exceeded the limit while riding (*OR* : 0.27, *p* *value* < 0.05). In other words, those who stayed within the speed limit had a lower likelihood of being in a road traffic crash than those who went over the limit (*OR* : 0.27, *p* *value* < 0.05). Finally, driving in rainy weather conditions increases the likelihood of being involved in a road traffic accident by approximately three times compared to driving in rainy, misty, or other weather conditions (*OR* : 3.102, *p* *value* < 0.05). In other words, drivers are more likely to be involved in a road traffic crash when the weather is rainy than when it is sunny, misty, or otherwise.

The Nagelkerke R-squared value (Table 3) of 0.556 suggests that approximately 55.7% of the variance in the dependent variable can be accounted for by the independent variables included in the model. Moreover, the Hosmer‒Lemeshow test indicates that the model adequately fits the data, as its *p* value is greater than 0.05.

The potential interactions have been explored by the chi-square test (Table 4). The chi-square and *p* values (in the parentheses) have been presented here. From the above table, it is observed that a total of thirty-seven pairs of interactions have been identified among the identified risk factors (Table 2). The factor of age is interconnected with the brand of motorcycle, motorcycle ownership, weekly working hours, and exceeding the speed limit. The other interactions are shown in Table 4. P–A value less than 0.05 indicates significant interactions.

## 4. Discussion

The study focuses on the effect of demographics, working history, lifestyle, behaviour, and miscellaneous factors, on the occurrence of RTCs among motorcyclists in Bangladesh. The results revealed that age significantly impacts RTCs, and young age (<20 years) motorcyclists had a substantially higher risk of RTCs than old age (>50 years). A previous study identifying the effect of age on RTCs found that age has a significant impact on RTCs [35]. Another study conducted in Malaysia on RTCs showed that younger motorcyclists were at a higher risk of facing RTCs than in another age group, especially between the ages of 10 and 16 [34]. Another identical research conducted in Dhaka, Bangladesh, found that over 50% of fatalities occurred in people under the age of 30 years [36]. The results showed that driving licenses significantly impact RTCs over one year among motorcyclists. According to the previous findings, a motorcyclist who does not have a riding license is unsafe while driving [32]. An earlier study identifying the effect of motorcycle licenses on motorcycle-car crashes found that drivers who have a motorcycle license are less likely to cause RTCs than drivers who do not [37]. Allison Daniello et al. reported that accident rates are lower in areas of the US where obtaining a license is more strictly regulated [38].

The study found that using a helmet is a risk factor for RTCs in motorcyclists. According to a previous survey by Abdi et al., wearing a helmet effectively reduces the severity of injuries sustained in motorcycle accidents in Africa [39]. Another study found that helmet-wearing is a significant factor negatively correlated with e-bike accident rates [40]. A similar outcome was observed in a previous study: a higher rate of fatalities among motorcyclists who did not wear helmets [41]. The motorcyclist's ability to avoid head injuries in traffic accidents was positively impacted by wearing a helmet [28, 42]. The analysis showed that weather conditions significantly impact RTCs among motorcyclists, and rainy days increase the accident rate more than other days. Another study conducted by Maidzadeh et al. found that weather significantly impacts accidents and severe injuries [43].

In contrast, rainfall reduced the probability of crashes across all severity levels [44]. From another contrasting result in China, the likelihood of fatal accidents was relatively lower on sunny days than under other weather conditions [45]. In addition, the study showed an association of exceeding car lanes on road boundaries with risk factors of RTCs among motorcyclists. From previous study findings, bicycle lanes significantly impacted road traffic crashes, indicating a reduction in RTCs [46]. Another study conducted in New York showed that painted bicycle lanes decreased the risk of injury by almost 90% [47].

Reckless overtaking of other cars while riding motorcycles is another significant positive factor for RTCs. More specifically, drinking while before driving is linked to a higher risk of being in a crash or falling [48]. Seva showed that drunk driving, not wearing a helmet, and underestimating the speed of an approaching vehicle when overtaking are all significant predictors of motorcycle accidents and severe injury [49]. The logistic regression results also demonstrated that exceeding the speed limit is a significant factor in motorcycle accidents. Moreover, a previous study found that substantial crash risk predictors were speeding violations [50]. According to another study with similar findings, higher speed limits were associated with more severe accidents and injuries in the Midwestern United States [51]. Another study conducted in Nigeria revealed that speeding and improper overtaking were significant factors in the rising rate of commercial motorcycle accidents [52]. In our study, smoking while driving was another significant factor in motorcycle accidents. A previous survey identifying the effect of smoking while driving on RTCs found that smoking while driving had a positive impact on motorcycle accidents [53]. Mallia discovered a significant positive relationship between smoking while driving and the number of accidents [54].

Moreover, this study revealed that working hours significantly impacted RTCs over one year among motorcyclists. The previous study's findings suggested that working full-time was linked to higher chances of being involved in a fatigue-related crash. Approximately 37% of all crashes that motorcycle taxi drivers reported had something to do with being fatigued while driving the motorcycle [55]. Peng et al. reported a significant effect of working hours on taxi crashes [56]. The findings of this study revealed that, as a developing country in Bangladesh, the RTCs are increasing day by day, specifically motorcycle traffic crashes. As a result, RTCs, injuries, and deaths occur every year and are widely rising. The results showed that the age of motorcyclists, presence of driving licenses, helmet use, weather conditions, traffic signals, exceeding car lanes on road boundaries, reckless overtaking, speed limit, smoking while driving, and working hours are the factors influencing RTCs among motorcyclists. It helps reduce motorcycle accidents and severe injuries by practising effective rules and techniques when driving a motorcycle.

### 4.1. Limitation of the Study

Although the results offer valuable perspectives on motorcycle-related road accidents in Noakhali, Bangladesh, it is important to approach generalizations to other settings or demographic groups with caution because of potential variations in sociocultural, economic, or infrastructural factors. However, the study has several limitations that need to be considered when interpreting the results. First, the sample size was relatively small, which may limit the generalizability of the findings to a wider population of motorcycle riders in Bangladesh or Noakhali. Second, the study relied on self-reported data, which may be subject to recall bias or social desirability bias. Third, the study did not investigate the role of other factors that may contribute to road traffic crashes, such as road conditions, weather, or vehicle maintenance.

## 5. Conclusion

The results of this study revealed that the overall reported prevalence of road traffic crashes involving motorcycle drivers was 68.66%, and the factors associated with traffic crashes included driving without a valid driving license, the young age (<20) of motorcyclists, driving in rainy weather, exceeding the speed limit, per-week working hours, smoking status, motorcycle ownership, the brand of motorcycle, and not wearing a helmet while driving. Based on the study's results, recommendations can be made to improve motorcycle safety in Noakhali, Bangladesh. These include implementing education and awareness programs to raise awareness of safe riding practices, the importance of helmet usage, and the dangers of traffic violations. The findings of this study can assist policymakers and concerned authorities in taking the essential steps to lessen road traffic crashes among motorcyclists in Bangladesh. Moreover, there are several opportunities for future research to build on the findings of this study, including more extensive studies exploring the prevalence of road traffic crashes among motorcycle drivers in other regions of Bangladesh or low- and middle-income countries.

## Figures and Tables

**Table 1:** Factors contributing to motorcycle accidents and rider characteristics in Noakhali, Bangladesh.

Variable	Category	*n*	%	No crash (*n*=126)		Crash (*n*=276)		*χ* ^2^	*P* value
				*n*_1_	%	*n*_2_	%		
Overall		402	100	126	31.34	276	68.66		

*Demographics*									
Age	<20	3	0.75	2	66.67	1	33.33	10.427	<0.05
	20–29	180	44.78	48	26.67	132	73.33		
	30–39	176	43.78	57	32.39	119	67.61		
	40–49	36	8.96	18	50.00	18	50.00		
	50–59	7	1.74	1	14.29	6	85.71		
	>60	0	0.00	0	0.00	0	0.00		

Marital status	Married	337	83.83	92	27.30	245	72.70	15.836	<0.001
	Unmarried	65	16.17	34	52.31	31	47.69		

Occupation	Student	45	11.19	25	55.56	20	44.44	13.804	<0.001
	Others	357	88.81	101	28.29	256	71.71		

Education level	High school	73	18.16	20	27.40	53	72.60	11.247	<0.05
	Above high school	291	72.39	85	29.21	206	70.79		
	Less than high school	38	9.45	21	55.26	17	44.74		

*Working history*									
Employment status	Part-time	262	65.17	66	25.19	196	74.81	13.233	<0.001
	Full-time	140	34.83	60	42.86	80	57.14		

Riding experience	<1	34	8.46	22	64.71	12	35.29	30.294	<0.001
	1 to 5	217	53.98	69	31.80	148	68.20		
	6 to 10	85	21.14	25	29.41	60	70.59		
	11 to 15	60	14.93	7	11.67	53	88.33		
	16 to 20	3	0.75	2	66.67	1	33.33		
	>21	3	0.75	1	33.33	2	66.67		

Motorcycle ownership	Inherits from relatives	127	31.59	66	51.97	61	48.03	36.7	<0.001
	Bought with own money	275	68.41	60	21.82	215	78.18		

Daily travel distance	<20 km	19	4.73	13	68.42	6	31.58	62.034	<0.001
	≥20 and <30 km	136	33.83	57	41.91	79	58.09		
	≥30 and <50 km	209	51.99	32	15.31	177	84.69		
	≥50 km	38	9.45	24	63.16	14	36.84		

Weekly working hours	<30 hours	22	5.47	13	59.09	9	40.91	61.33	<0.001
	≥40 and <50 hours	286	71.14	57	19.93	229	80.07		
	≥50 and <60 hours	53	13.18	29	54.72	24	45.28		
	≥60 hours	41	10.20	27	65.85	14	34.15		

*Riders' lifestyle and behaviour*									
Do you talk on the phone while riding motorcycle? If yes, how often?	Sometimes	264	65.67	108	40.91	156	59.09	32.944	<0.001
	Often	135	33.58	18	13.33	117	86.67		
	Regularly	3	0.75	0	0.00	3	100.00		

Smoking status	Never	240	59.70	80	33.33	160	66.67	7.888	<0.05
	Sometimes	46	11.44	20	43.48	26	56.52		
	Regularly	116	28.86	26	22.41	90	77.59		
	Yes	387	96.27	116	29.97	271	70.03		

Smoke while driving?	No	255	63.43	110	43.14	145	56.86	45.076	<0.001
	Yes	147	36.57	16	10.88	131	89.12		

*Miscellaneous*									
Use helmet?	No	198	49.25	25	12.63	173	87.37	63.519	<0.001
	Yes	229	56.97	126	55.02	103	44.98		

Motorcycle's brand	Honda	86	21.39	37	43.02	49	56.98	31.334	<0.001
	Yamaha	75	18.66	33	44.00	42	56.00		
	Suzuki	141	35.07	20	14.18	121	85.82		
	Others	100	24.88	36	36.00	64	64.00		

Have riding license?	No	72	17.91	48	66.67	24	33.33	50.856	<0.001
	Yes	330	82.09	78	23.64	252	76.36		

Exceed the speed limit?	No	290	72.14	117	40.34	173	59.66	39.193	<0.001
	Yes	112	27.86	9	8.04	103	91.96		

Red-light running?	No	15	3.73	10	66.67	5	33.33	9.034	<0.05
	Yes	387	96.27	116	29.97	271	70.03		

*n*:total number of respondents, *n*_1_:number of noncrashers in the past year, *n*_2_:crash victims in the past year.

**Table 2:** Influence of various factors related to motorcycle riders on the likelihood of road traffic crashes (logistic regression model).

Variable	Category	Coefficient	Std. Error	*P* value	OR	95% C.I. for OR	
						Lower	Upper
*Demographics*							
Age	<20	−6.318	2.971	0.033	0.002	0	0.609
	21–29	−2.135	1.502	0.155	0.118	0.006	2.248
	30–39	−2.352	1.488	0.114	0.095	0.005	1.759
	40–49	−2.86	1.54	0.063	0.057	0.003	1.172
	>50	Ref	Ref	Ref	Ref	Ref	Ref

*Working history*							
Motorcycle ownership	Inherits from relatives	−0.879	0.344	0.011	0.415	0.211	0.815
	Bought with own money	Ref	Ref	Ref	Ref	Ref	Ref

Weekly working hours	<30 hours	0.702	0.74	0.342	2.019	0.473	8.605
	≥40 and <50 hours	1.414	0.672	0.035	4.113	1.102	15.347
	≥50 and <60 hours	0.969	0.705	0.169	2.635	0.662	10.483
	≥60 hours	Ref	Ref	Ref	Ref	Ref	Ref

*Riders' lifestyle and behaviours*							
Smoking status	Never	−0.664	0.379	0.08	0.515	0.245	1.081
	Sometimes	−1.258	0.514	0.014	0.284	0.104	0.779
	Regularly	Ref	Ref	Ref	Ref	Ref	Ref

*Miscellaneous*							
Use helmet?	No	1.231	0.358	0.001	3.423	1.699	6.899
	Yes	Ref	Ref	Ref	Ref	Ref	Ref

Motorcycle's brand	Honda	0.16	0.422	0.704	1.174	0.513	2.683
	Yamaha	0.096	0.441	0.827	1.101	0.464	2.61
	Suzuki	1.009	0.433	0.02	2.744	1.175	6.406
	Others	Ref	Ref	Ref	Ref	Ref	Ref

Have riding license?	No	−1.092	0.374	0.004	0.335	0.161	0.699
	Yes	Ref	Ref	Ref	Ref	Ref	Ref

Exceed speed limit?	No	−1.308	0.561	0.02	0.27	0.09	0.812
	Yes	Ref	Ref	Ref	Ref	Ref	Ref

Weather condition?	Sunny	0.198	0.404	0.625	1.219	0.552	2.691
	Rainy	1.132	0.51	0.026	3.102	1.142	8.423
	Misty	0.036	0.451	0.937	1.036	0.428	2.509
	Others	Ref	Ref	Ref	Ref	Ref	Ref

Constant		4.529	1.985	0.023	92.625		

OR=odds ratio and CI=confidence interval.

**Table 3:** Model summary and Hosmer–Lemeshow test.

Nagelkerke *R* square	Hosmer–Lemeshow test		
	Chi-square	df	*P* value
0.556	10.727	8	0.218

**Table 4:** Potential interactions between the identified risk factors.

	Age	UH	MB	MO	SS	WWH	ESL	WC	DDL
Age		9.043 (0.06)	24.607 (0.017)	11.411 (0.022)	8.622 (0.375)	37.503 (0.000)	13.105 (0.11)	6.29 (0.901)	9.334 (0.053)
UH	9.043 (0.06)		12.17 (0.017)	27.761 (0.000)	11.447 (0.003)	53.778 (0.000)	12.418 (0.000)	2.893 (0.408)	18.346 (0.000)
MB	24.607 (0.017)	12.17 (0.007)		10.946 (0.012)	2.734 (0.841)	31.107 (0.000)	17.362 (0.001)	1.395 (0.998)	17.186 (0.001)
MO	11.411 (0.022)	27.761 (0.000)	10.946 (0.012)		5.219 (0.074)	23.944 (0.000)	5.042 (0.025)	0.578 (0.901)	35.364 (0.000)
SS	8.622 (0.375)	11.447 (0.003)	2.734 (0.841)	5.219 (0.074)		11.632 (0.071)	3.356 (0.187)	9.47 (0.149)	1.145 (0.564)
WWH	37.503 (0.000)	53.778 (0.000)	31.107 (0.000)	23.944 (0.000)	11.632 (0.071)		25.037 (0.000)	8.822 (0.454)	17.978 (0.000)
ESL	13.105 (0.011)	12.418 (0.000)	17.362 (0.001)	5.042 (0.025)	3.356 (0.187)	25.037 (0.000)		2.942 (0.401)	21.712 (0.000)
WC	6.29 (0.901)	2.893 (0.408)	1.395 (0.998)	0.578 (0.901)	9.47 (0.149)	8.822 (0.454)	2.942 (0.401)		3.508 (0.32)
DDL	9.334 (0.053)	18.346 (0.000)	17.186 (0.001)	35.364 (0.000)	1.145 (0.564)	17.978 (0.000)	21.712 (0.000)	3.508 (0.32)	

DDL: do you have a driver's license?, WC: weather condition, ESL: exceeding the speed limit, WWH: weekly working hours, SS: smoking status, MO: motorcycle ownership, MB: brand of motorcycle, UH: use of helmet.

## Data Availability

The dataset for this study is publicly available at https://data.mendeley.com/datasets/32hc3wydkv/.

## Supplementary Materials

Supplementary Materials Questionnaire (pdf). Raw data (pdf).
