# Supplementary material for: Analyzing the Prevalence of and Factors Associated with Road Traffic Crashes (RTCs) among Motorcyclists in Bangladesh
Source: ScientificWorldJournal. 2024 May 9;2024:7090576. doi: 10.1155/2024/7090576 (PMC11098599; doi:10.1155/2024/7090576)
Supplement: Supplementary Materials — Questionnaire (pdf). Raw data (pdf). [file 7090576.f1.zip › Questionnaire.pdf]

Questionnaire on "Analysing the prevalence and associated factors with road traffic crashes among motorcycle riders in Bangladesh"

1. **Gender**
    - Male
    - Female

2. **Living place**
    - Rural
    - Urban

3. **Age**
    - <20
    - 20-29
    - 30-39
    - 40-49
    - 50-59
    - >60

4. **Riding experience (in year)**
    - <1
    - 1 to 5
    - 6 to 10
    - 11 to 15
    - 16 to 20
    - >21

5. **Marital status**
    - Married
    - Unmarried

6. **Occupation**
    - Student
    - Others

7. **Education level**
    - High school
    - Above high school
    - Less than high school

8. **Employment status**
    - Part-time
    - Full-time

9. Motorcycle's brand

- Honda
- Yamaha
- Suzuki

10. Is this registered motorcycle?

- No
- Yes

11. Do you have riding license?

- No
- Yes

12. Motorcycle ownership

- Inherits from relatives
- Bought with own money

13. Daily travel distance (in km.)

- <20
- ≥ 20 and <30
- ≥ 30 and <50
- ≥ 50

14. Weekly working hours

- <30
- ≥ 40 and <50
- ≥ 50 and <60
- ≥ 60

15. Have you faced any motorcycle traffic crashes over the last one-year period?

- No
- Yes

16. Any injury caused by motorcycle traffic crashes over the last one-year period?

- No
- Yes

17. Accident severity types that you faced over the last one-year period?

- Severe
- Moderate
- Minor

18. Weather condition while accident took place

- Sunny
- Rainy
- Misty

- Others

19. Do you talk on the phone while riding motorcycle? If yes, how often?
- Sometimes
- Often
- Regularly

20. Drinking status
- Never
- Sometimes
- Regularly

21. Smoking status
- Never
- Sometimes
- Regularly

22. Turn signal neglect?
- No
- Yes

23. Encroach car lanes?
- No
- Yes

24. Exceed speed limit?
- No
- Yes

25. Red-light running?
- No
- Yes

26. Carry more than one passenger?
- No
- Yes

27. Smoke while driving
- Yes
- No

28. Use helmet?
- No
- Yes

29. Reckless overtaking?

- No
- Yes

30. Drink driving?

- No
- yes
