# Supplementary material for: Analyzing the Prevalence of and Factors Associated with Road Traffic Crashes (RTCs) among Motorcyclists in Bangladesh
Source: ScientificWorldJournal. 2024 May 9;2024:7090576. doi: 10.1155/2024/7090576 (PMC11098599; doi:10.1155/2024/7090576)
Supplement: Supplementary Materials — Questionnaire (pdf). Raw data (pdf). [file 7090576.f1.zip › Raw data.pdf]

1. Gender	2. Living place	3. Age	4. Riding experience (in year)	5. Marital status
Male	Rural	20-29	1-May	Unmarried
Male	Rural	30-39	1-May	Married
Male	Rural	20-29	1-May	Unmarried
Male	Rural	30-39	1-May	Married
Male	Rural	20-29	Nov-15	Unmarried
Male	Rural	50-59	>21	Married
Male	Rural	30-39	6-Oct	Married
Male	Rural	20-29	<1 year	Unmarried
Male	Rural	30-39	Nov-15	Married
Male	Rural	30-39	Nov-15	Married
Male	Rural	20-29	1-May	Unmarried
Male	Rural	30-39	1-May	Unmarried
Male	Rural	20-29		Unmarried
Male	Rural	20-29	1-May	Unmarried
Male	Rural	30-39	6-Oct	Married
Male	Rural	20-29	6-Oct	Married
Male	Rural	<20	1-May	Unmarried
Male	Rural	30-39	1-May	Married
Male	Rural	20-29	<1 year	Unmarried
Male	Rural	30-39	1-May	Married
Male	Rural	40-49	6-Oct	Married
Male	Rural	20-29	1-May	Unmarried
Male	Rural	40-49	Nov-15	Married
Male	Rural	30-39	1-May	Married
Female	Rural	20-29	<1 year	Unmarried
Female		30-39	1-May	Married
Male	Rural	30-39	1-May	Married
Male	Rural	30-39	6-Oct	Married
Female	Rural	20-29	<1 year	Married
Male	Rural	40-49	Nov-15	Married
Male	Rural	30-39	6-Oct	Married
Male	Rural	20-29	<1 year	Unmarried
Male	Rural	20-29	<1 year	Married
Male	Rural	20-29	<1 year	Unmarried
Male	Rural	30-39	1-May	Married
Male	Rural	30-39	6-Oct	Married
Male	Rural	50-59	Nov-15	Married
Male	Rural	20-29	6-Oct	Married
Male	Rural	40-49	6-Oct	Married
Male	Rural	40-49	6-Oct	Married
Male	Rural	30-39	6-Oct	Married
Male	Rural	30-39	6-Oct	Married
Male	Rural	40-49	16-20	Married
Male	Rural	50-59	>21	Married
Male	Rural	40-49	16-20	Married
Male	Rural	30-39	Nov-15	Married
Male	Rural	20-29	<1 year	Unmarried

Male	Rural	20-29	1-May	Unmarried
Male	Rural	20-29	6-Oct	Married
Male	Rural	20-29	1-May	Unmarried
Male	Rural	20-29	<1 year	Unmarried
Male	Rural	30-39	1-May	Married
Male	Rural	40-49	6-Oct	Married
Male	Rural	20-29	<1 year	Unmarried
Male	Rural	40-49	1-May	Married
Male	Rural	30-39	1-May	Married
Female	Rural	20-29	1-May	Married
Male	Rural	30-39	1-May	Married
Male	Rural	20-29	<1 year	Unmarried
Male	Rural	40-49	6-Oct	Married
Male	Rural	30-39	1-May	Married
Male	Rural	50-59	Nov-15	Married
	Rural	20-29	1-May	Married
Male	Rural	20-29	1-May	Unmarried
Male	Rural	20-29	1-May	Married
Male	Rural	30-39	6-Oct	Married
Male	Rural	30-39	6-Oct	Married
Male	Rural	30-39	1-May	Married
Male	Rural	30-39	6-Oct	Married
Male	Rural	20-29	1-May	Unmarried
Male	Rural	20-29	1-May	Unmarried
Male	Rural	20-29	<1 year	Married
Male	Rural	20-29	1-May	Married
Male	Rural	30-39	1-May	Married
Male	Rural	30-39	1-May	Married
Male	Rural	30-39	6-Oct	Married
Female	Rural	20-29	1-May	Unmarried
Male	Rural	20-29	1-May	Unmarried
Male	Rural	30-39	1-May	Married
Male	Rural	30-39	1-May	Married
Male	Rural	20-29	<1 year	Unmarried
Female	Rural	20-29	<1 year	Unmarried
Male	Rural	20-29	1-May	Unmarried
Male	Rural	30-39	1-May	Married
Male	Rural	30-39	1-May	Married
Male	Rural	40-49	6-Oct	Married
Male	Rural	20-29	<1 year	Unmarried
Female	Rural	30-39	1-May	Married
Male	Rural	30-39	1-May	Married
Female	Rural	20-29	<1 year	Married
Male	Rural	30-39	1-May	Married
Male	Rural	40-49	Nov-15	Married
Male	Rural	30-39	6-Oct	Married
Male	Rural	30-39	1-May	Unmarried
Male	Rural	20-29	<1 year	Unmarried
Male	Rural	30-39	1-May	Married
Male	Rural	40-49	6-Oct	Married

Male	Rural	20-29	1-May	Married
Male	Rural	40-49	1-May	Married
Male	Rural	30-39	1-May	Married
Male	Rural	30-39	1-May	Unmarried
Female	Rural	30-39	1-May	Married
Male	Rural	30-39	1-May	Married
Female	Rural	20-29	>21	Unmarried
Male	Rural	20-29	1-May	Married
Male	Rural	20-29	1-May	Married
Male	Rural	20-29	1-May	Married
Male	Rural	30-39	1-May	Unmarried
Male	Rural	20-29	<1 year	Unmarried
Male	Rural	30-39	1-May	Unmarried
Male	Rural	30-39	1-May	Married
Male	Rural	40-49	1-May	Married
Male	Rural	30-39	1-May	Married
Male	Rural	30-39	1-May	Married
Male	Rural	20-29	<1 year	Married
Male	Rural	30-39	1-May	Married
Male	Rural	20-29	<1 year	Married
Male	Rural	40-49	Nov-15	Married
Male	Rural	20-29	1-May	Unmarried
Female	Rural	20-29	1-May	Married
Male	Rural	30-39	1-May	Married
Male	Rural	30-39	1-May	Married
Male	Rural	20-29	<1 year	Unmarried
Male	Rural	20-29	1-May	Unmarried
Male	Rural	30-39	1-May	Married
Female	Rural	30-39	1-May	Married
Male	Rural	30-39	Nov-15	Married
Male	Rural	20-29	1-May	Unmarried
Male	Rural	40-49	6-Oct	Married
Male	Rural	30-39	1-May	Married
Male	Rural	20-29	<1 year	Unmarried
Male	Rural	40-49	1-May	Married
Male	Rural	30-39	1-May	Married
Male	Rural	30-39	6-Oct	Married
Male	Rural	20-29	1-May	Married
Female	Rural	30-39	<1 year	Married
Male	Rural	30-39	1-May	Married
Male	Rural	20-29	<1 year	Unmarried
Male	Rural	20-29	1-May	Married
Male	Rural	30-39	1-May	Married
Male	Rural	30-39	<1 year	Married
Male	Rural	30-39	1-May	Married
Male	Rural	30-39	1-May	Married
Male	Rural	20-29	1-May	Unmarried
Male	Rural	30-39	6-Oct	Married
Female	Rural	20-29	1-May	Unmarried
Male	Rural	30-39	6-Oct	Married

Male	Rural	20-29	6-Oct	Unmarried
Male	Rural	40-49	16-20	Married
Male	Rural	20-29	1-May	Unmarried
Male	Rural	30-39	1-May	Married
Male	Rural	30-39	6-Oct	Married
Female	Rural	20-29	<1 year	Unmarried
Male	Rural	30-39	1-May	Married
Male	Rural	20-29	1-May	Married
Male	Rural	30-39	1-May	Married
Male	Rural	20-29	<1 year	Unmarried
Male	Rural	30-39	6-Oct	Married
Male	Rural	30-39	1-May	Married
Male	Rural	20-29	1-May	Unmarried
Male	Rural	30-39	1-May	Married
Female	Rural	20-29	1-May	Married
Male	Rural	40-49	6-Oct	Married
Male	Rural	30-39	1-May	Married
Male	Rural	20-29	<1 year	Unmarried
Male	Rural	30-39	<1 year	Married
Male	Rural	50-59	6-Oct	Married
Male	Rural	50-59	6-Oct	Married
Male	Rural	50-59	6-Oct	Married
Male	Rural	20-29	1-May	Married
Male	Rural	20-29	1-May	Married
Male	Rural	30-39	6-Oct	Married
Male	Rural	40-49	1-May	Married
Male	Rural	30-39	1-May	Married
Male	Rural	40-49	6-Oct	Married
Male	Rural	20-29	6-Oct	Married
Male	Rural	30-39	6-Oct	Married
Male	Rural	20-29	1-May	Unmarried
Female	Rural	20-29	1-May	Unmarried
Male	Rural	40-49	Nov-15	Married
Male	Rural	30-39	1-May	Married
Male	Rural	20-29	1-May	Unmarried
Male	Rural	30-39	6-Oct	Married
Male	Rural	30-39	1-May	Married
Male	Rural	20-29	<1 year	Married
Male	Rural	40-49	6-Oct	Married
Male	Rural	40-49	1-May	Married
Female	Rural	20-29	<1 year	Unmarried
Male	Rural	40-49	1-May	Married
Male	Rural	20-29	<1 year	Unmarried
Male	Rural	20-29	<1 year	Unmarried
Male	Rural	30-39	1-May	Married
Male	Rural	20-29	1-May	Unmarried
Male	Rural	30-39	1-May	Married
Male	Rural	30-39	1-May	Married
Female	Rural	30-39	1-May	Married
Male	Rural	40-49	6-Oct	Married

Male	Rural	30-39	1-May	Married
Male	Rural	30-39	1-May	Married
Male	Rural	20-29	1-May	Married
Male	Rural	30-39	1-May	Married
Male	Rural	30-39	1-May	Married
Male	Rural	30-39	6-Oct	Married
Male	Rural	20-29	1-May	Married
Male	Rural	30-39	1-May	Married
Male	Rural	30-39	6-Oct	Married
Male	Rural	30-39	1-May	Married
Male	Rural	30-39	Nov-15	Married
Female	Rural	30-39	Nov-15	Married
Male	Rural	30-39	Nov-15	Married
Male	Rural	20-29	1-May	Married
Male	Rural	30-39	1-May	Married
Male	Rural	30-39	1-May	Married
Male	Rural	30-39	1-May	Married
Male	Rural	30-39	1-May	Married
Male	Rural	30-39	1-May	Married
Male	Rural	20-29	1-May	Married
Male	Rural	20-29	1-May	Unmarried
Female	Rural	30-39	1-May	Married
Female	Rural	20-29	1-May	Unmarried
Male	Rural	20-29	Nov-15	Married
Male	Rural	40-49	Nov-15	Married
Male	Rural	30-39	Nov-15	Married
Male	Rural	30-39	Nov-15	Married
Male	Rural	20-29	1-May	Unmarried
Male	Rural	30-39	6-Oct	Married
Male	Rural	20-29	1-May	Unmarried
Male	Rural	20-29	1-May	Married
Male	Rural	30-39	Nov-15	Married
Male	Rural	30-39	1-May	Married
Male	Rural	30-39	1-May	Married
Male	Rural	20-29	Nov-15	Married
Male	Rural	30-39	1-May	Married
Male	Rural	40-49	Nov-15	Married
Male	Rural	20-29	1-May	Married
Male	Rural	20-29	<1 year	Married
Male	Rural	30-39	Nov-15	Married
Male	Rural	30-39	1-May	Married
Male	Rural	20-29	1-May	Married
Male	Rural	20-29	6-Oct	Married
Male	Rural	20-29	6-Oct	Married
Male	Rural	30-39	Nov-15	Married
Male	Rural	30-39	Nov-15	Married
Male	Rural	30-39	1-May	Married
Male	Rural	20-29	1-May	Married
Male	Rural	30-39	Nov-15	Married
Male	Rural	20-29	1-May	Unmarried

Female	Rural	20-29	1-May	Married
Male	Rural	20-29	6-Oct	Married
Male	Rural	20-29	6-Oct	Married
Male	Rural	30-39	1-May	Married
Male	Rural	20-29	6-Oct	Married
Male	Rural	30-39	1-May	Married
Male	Rural	30-39	Nov-15	Married
Male	Rural	30-39	1-May	Married
Male	Rural	30-39	1-May	Married
Male	Rural	30-39	1-May	Married
Male	Rural	30-39	1-May	Married
Male	Rural	20-29	1-May	Married
Male	Rural	30-39	Nov-15	Married
Female	Rural	30-39	Nov-15	Married
Female	Rural	20-29	6-Oct	Married
Male	Rural	30-39	Nov-15	Married
Female	Rural	30-39	1-May	Married
Male	Rural	20-29	1-May	Married
Male	Rural	20-29	1-May	Married
Male	Rural	20-29	1-May	Married
Male	Rural	20-29	1-May	Married
Male	Rural	20-29	6-Oct	Married
Female	Rural	30-39	Nov-15	Married
Male	Rural	20-29	1-May	Married
Female	Rural	20-29	1-May	Married
Female	Rural	40-49	Nov-15	Married
Male	Rural	30-39	6-Oct	Married
Female	Rural	20-29	6-Oct	Married
Male	Rural	30-39	Nov-15	Married
Male	Rural	20-29	Nov-15	Married
Male	Rural	30-39	1-May	Married
Male	Rural	20-29	6-Oct	Married
Male	Rural	20-29	Nov-15	Married
Male	Rural	20-29	6-Oct	Married
Male	Rural	30-39	Nov-15	Married
Female	Rural	20-29	1-May	Married
Male	Rural	30-39	6-Oct	Married
Female	Rural	30-39	1-May	Married
Male	Rural	30-39	Nov-15	Married
Female	Rural	20-29	Nov-15	Married
Male	Rural	20-29	6-Oct	Married
Male	Rural	20-29	1-May	Married
Male	Rural	<20	1-May	Married
Female	Rural	20-29	1-May	Married
Male	Rural	20-29	1-May	Married
Female	Rural	20-29	6-Oct	Married
Male	Rural	30-39	Nov-15	Married
Male	Rural	30-39	6-Oct	Married
Male	Rural	20-29	1-May	Married
Female	Rural	20-29	1-May	Married

Male	Rural	20-29	6-Oct	Married
Female	Rural	20-29	6-Oct	Married
Male	Rural	20-29	1-May	Married
Male	Rural	30-39	Nov-15	Married
Male	Rural	20-29	1-May	Married
Male	Rural	30-39	6-Oct	Married
Male	Rural	20-29	1-May	Married
Female	Rural	20-29	Nov-15	Married
Male	Rural	30-39	Nov-15	Married
Female	Rural	20-29	1-May	Married
Male	Rural	20-29	1-May	Married
Male	Rural	20-29	1-May	Married
Male	Rural	30-39	1-May	Married
Male	Rural	20-29	1-May	Married
Male	Rural	30-39	1-May	Married
Male	Rural	30-39	Nov-15	Married
Male	Rural	20-29	1-May	Married
Male	Rural	30-39	6-Oct	Married
Female	Rural	30-39	1-May	Married
Male	Rural	30-39	1-May	Married
Female	Rural	30-39	1-May	Married
Female	Rural	20-29	1-May	Married
Male	Rural	20-29	1-May	Married
Male	Rural	30-39	Nov-15	Married
Female	Rural	20-29	1-May	Married
Male	Rural	20-29	1-May	Married
Male	Rural	20-29	1-May	Married
Male	Rural	30-39	1-May	Married
Female	Rural	20-29	1-May	Married
Male	Rural	20-29	1-May	Married
Male	Rural	20-29	1-May	Married
Male	Rural	30-39	6-Oct	Married
Male	Rural	30-39	1-May	Married
Male	Rural	30-39	1-May	Married
Male	Rural	30-39	Nov-15	Married
Male	Rural	30-39	6-Oct	Married
Male	Rural	30-39	6-Oct	Married
Male	Rural	30-39	Nov-15	Married
Female	Rural	30-39	Nov-15	Married
Male	Rural	30-39	1-May	Married
Female	Rural	20-29	1-May	Married
Male	Rural	20-29	1-May	Married
Male	Rural	20-29	1-May	Unmarried
Male	Rural	20-29	1-May	Married
Male	Rural	30-39	6-Oct	Married
Male	Rural	20-29	1-May	Unmarried
Female	Rural	20-29	1-May	Married
Male	Rural	20-29	1-May	Married
Male	Rural	20-29	6-Oct	Married
Male	Rural	30-39	Nov-15	Married

Male	Rural	20-29	1-May	Married
Male	Rural	20-29	1-May	Married
Male	Rural	20-29	1-May	Married
Male	Rural	30-39	1-May	Married
Male	Rural	20-29	6-Oct	Married
Male	Rural	30-39	6-Oct	Married
Male	Rural	30-39	1-May	Married
Male	Rural	20-29	1-May	Married
Male	Rural	20-29	Nov-15	Married
Male	Rural	20-29	1-May	Married
Male	Rural	20-29	Nov-15	Married
Male	Rural	20-29	6-Oct	Married
Male	Rural	30-39	1-May	Married
Female	Rural	20-29	1-May	Married
Male	Rural	20-29	1-May	Married
Male	Rural	30-39	1-May	Married
Male	Rural	30-39	1-May	Married
Male	Rural	40-49	6-Oct	Married
Male	Rural	30-39	Nov-15	Married
Male	Rural	30-39	6-Oct	Married
Male	Rural	20-29	Nov-15	Married
Male	Rural	30-39	6-Oct	Married
Male	Rural	20-29	Nov-15	Married
Male	Rural	20-29	1-May	Married
Male	Rural	20-29	1-May	Married
Male	Rural	20-29	1-May	Married
Male	Rural	20-29	6-Oct	Married
Male	Rural	30-39	6-Oct	Married
Female	Rural	20-29	Nov-15	Married
Male	Rural	20-29	1-May	Married
Male	Rural	40-49	Nov-15	Married
Male	Rural	30-39	Nov-15	Married
Male	Rural	20-29	1-May	Married
Male	Rural	20-29	6-Oct	Married
Male	Rural	20-29	6-Oct	Married
Male	Rural	20-29	1-May	Married
Male	Rural	20-29	1-May	Married
Male	Rural	20-29	1-May	Married
Male	Rural	20-29	6-Oct	Married
Female	Rural	20-29	1-May	Married
Female	Rural	20-29	1-May	Married
Male	Rural	40-49	Nov-15	Married
Male	Rural	30-39	6-Oct	Married
Male	Rural	20-29	1-May	Married
Female	Rural	20-29	1-May	Married
Male	Rural	40-49	Nov-15	Married
Male	Rural	30-39	6-Oct	Married
Male	Rural	40-49	6-Oct	Married
Male	Rural	40-49	6-Oct	Married
Male	Rural	30-39	6-Oct	Married

Male	Rural	30-39	Nov-15	Married
Male	Rural	30-39	1-May	Married
Male	Rural	20-29	6-Oct	Married
Male	Rural	30-39	1-May	Married

6. Occupation

Student
Others (including workers, office staff, graduated students who are looking for work)
Student
Others (including workers, office staff, graduated students who are looking for work)
Student
Others (including workers, office staff, graduated students who are looking for work)
Others (including workers, office staff, graduated students who are looking for work)
Student
Others (including workers, office staff, graduated students who are looking for work)
Others (including workers, office staff, graduated students who are looking for work)
Student
Others (including workers, office staff, graduated students who are looking for work)
Student
Student
Others (including workers, office staff, graduated students who are looking for work)

Others (including workers, office staff, graduated students who are looking for work)
Student
Others (including workers, office staff, graduated students who are looking for work)
Student
Others (including workers, office staff, graduated students who are looking for work)
Others (including workers, office staff, graduated students who are looking for work)
Others (including workers, office staff, graduated students who are looking for work)
Others (including workers, office staff, graduated students who are looking for work)
Others (including workers, office staff, graduated students who are looking for work)
Student
Others (including workers, office staff, graduated students who are looking for work)
Others (including workers, office staff, graduated students who are looking for work)
Others (including workers, office staff, graduated students who are looking for work)
Others (including workers, office staff, graduated students who are looking for work)
Others (including workers, office staff, graduated students who are looking for work)
Others (including workers, office staff, graduated students who are looking for work)
Student
Others (including workers, office staff, graduated students who are looking for work)
Student
Others (including workers, office staff, graduated students who are looking for work)
Others (including workers, office staff, graduated students who are looking for work)
Others (including workers, office staff, graduated students who are looking for work)
Others (including workers, office staff, graduated students who are looking for work)
Others (including workers, office staff, graduated students who are looking for work)
Others (including workers, office staff, graduated students who are looking for work)
Others (including workers, office staff, graduated students who are looking for work)
Others (including workers, office staff, graduated students who are looking for work)
Others (including workers, office staff, graduated students who are looking for work)
Others (including workers, office staff, graduated students who are looking for work)
Others (including workers, office staff, graduated students who are looking for work)
Others (including workers, office staff, graduated students who are looking for work)
Student

Student
Others (including workers, office staff, graduated students who are looking for work)
Others (including workers, office staff, graduated students who are looking for work)
Student
Others (including workers, office staff, graduated students who are looking for work)
Others (including workers, office staff, graduated students who are looking for work)
Student
Others (including workers, office staff, graduated students who are looking for work)
Others (including workers, office staff, graduated students who are looking for work)
Others (including workers, office staff, graduated students who are looking for work)
Others (including workers, office staff, graduated students who are looking for work)
Others (including workers, office staff, graduated students who are looking for work)
Others (including workers, office staff, graduated students who are looking for work)
Others (including workers, office staff, graduated students who are looking for work)
Others (including workers, office staff, graduated students who are looking for work)
Others (including workers, office staff, graduated students who are looking for work)
Student
Others (including workers, office staff, graduated students who are looking for work)
Others (including workers, office staff, graduated students who are looking for work)
Others (including workers, office staff, graduated students who are looking for work)
Others (including workers, office staff, graduated students who are looking for work)
Others (including workers, office staff, graduated students who are looking for work)
Others (including workers, office staff, graduated students who are looking for work)
Others (including workers, office staff, graduated students who are looking for work)
Student
Others (including workers, office staff, graduated students who are looking for work)
Others (including workers, office staff, graduated students who are looking for work)
Others (including workers, office staff, graduated students who are looking for work)
Others (including workers, office staff, graduated students who are looking for work)
Student
Student
Others (including workers, office staff, graduated students who are looking for work)
Others (including workers, office staff, graduated students who are looking for work)
Student
Student
Others (including workers, office staff, graduated students who are looking for work)
Others (including workers, office staff, graduated students who are looking for work)
Others (including workers, office staff, graduated students who are looking for work)
Others (including workers, office staff, graduated students who are looking for work)
Others (including workers, office staff, graduated students who are looking for work)
Others (including workers, office staff, graduated students who are looking for work)
Others (including workers, office staff, graduated students who are looking for work)
Others (including workers, office staff, graduated students who are looking for work)
Others (including workers, office staff, graduated students who are looking for work)
Others (including workers, office staff, graduated students who are looking for work)
Others (including workers, office staff, graduated students who are looking for work)
Others (including workers, office staff, graduated students who are looking for work)
Student
Others (including workers, office staff, graduated students who are looking for work)
Others (including workers, office staff, graduated students who are looking for work)

Others (including workers, office staff, graduated students who are looking for work)
Others (including workers, office staff, graduated students who are looking for work)
Others (including workers, office staff, graduated students who are looking for work)
Others (including workers, office staff, graduated students who are looking for work)
Others (including workers, office staff, graduated students who are looking for work)
Others (including workers, office staff, graduated students who are looking for work)
Student
Others (including workers, office staff, graduated students who are looking for work)
Others (including workers, office staff, graduated students who are looking for work)
Others (including workers, office staff, graduated students who are looking for work)
Others (including workers, office staff, graduated students who are looking for work)
Others (including workers, office staff, graduated students who are looking for work)
Others (including workers, office staff, graduated students who are looking for work)
Others (including workers, office staff, graduated students who are looking for work)
Others (including workers, office staff, graduated students who are looking for work)
Others (including workers, office staff, graduated students who are looking for work)
Others (including workers, office staff, graduated students who are looking for work)
Student
Others (including workers, office staff, graduated students who are looking for work)
Others (including workers, office staff, graduated students who are looking for work)
Others (including workers, office staff, graduated students who are looking for work)
Student
Others (including workers, office staff, graduated students who are looking for work)
Others (including workers, office staff, graduated students who are looking for work)
Others (including workers, office staff, graduated students who are looking for work)
Student
Others (including workers, office staff, graduated students who are looking for work)
Others (including workers, office staff, graduated students who are looking for work)
Others (including workers, office staff, graduated students who are looking for work)
Others (including workers, office staff, graduated students who are looking for work)
Student
Others (including workers, office staff, graduated students who are looking for work)
Others (including workers, office staff, graduated students who are looking for work)
Student
Others (including workers, office staff, graduated students who are looking for work)
Others (including workers, office staff, graduated students who are looking for work)
Others (including workers, office staff, graduated students who are looking for work)
Others (including workers, office staff, graduated students who are looking for work)

Others (including workers, office staff, graduated students who are looking for work)
Others (including workers, office staff, graduated students who are looking for work)
Others (including workers, office staff, graduated students who are looking for work)
Others (including workers, office staff, graduated students who are looking for work)
Others (including workers, office staff, graduated students who are looking for work)
Others (including workers, office staff, graduated students who are looking for work)
Others (including workers, office staff, graduated students who are looking for work)
Student
Others (including workers, office staff, graduated students who are looking for work)
Student
Others (including workers, office staff, graduated students who are looking for work)

Others (including workers, office staff, graduated students who are looking for work)
Others (including workers, office staff, graduated students who are looking for work)
Student
Others (including workers, office staff, graduated students who are looking for work)
Others (including workers, office staff, graduated students who are looking for work)
Student
Others (including workers, office staff, graduated students who are looking for work)
Others (including workers, office staff, graduated students who are looking for work)
Others (including workers, office staff, graduated students who are looking for work)
Student
Others (including workers, office staff, graduated students who are looking for work)
Others (including workers, office staff, graduated students who are looking for work)
Others (including workers, office staff, graduated students who are looking for work)
Others (including workers, office staff, graduated students who are looking for work)
Others (including workers, office staff, graduated students who are looking for work)
Others (including workers, office staff, graduated students who are looking for work)
Others (including workers, office staff, graduated students who are looking for work)
Student
Others (including workers, office staff, graduated students who are looking for work)
Others (including workers, office staff, graduated students who are looking for work)
Others (including workers, office staff, graduated students who are looking for work)
Others (including workers, office staff, graduated students who are looking for work)
Others (including workers, office staff, graduated students who are looking for work)
Others (including workers, office staff, graduated students who are looking for work)
Others (including workers, office staff, graduated students who are looking for work)
Others (including workers, office staff, graduated students who are looking for work)
Others (including workers, office staff, graduated students who are looking for work)
Others (including workers, office staff, graduated students who are looking for work)
Others (including workers, office staff, graduated students who are looking for work)
Others (including workers, office staff, graduated students who are looking for work)
Student
Student
Others (including workers, office staff, graduated students who are looking for work)
Others (including workers, office staff, graduated students who are looking for work)
Student
Others (including workers, office staff, graduated students who are looking for work)
Others (including workers, office staff, graduated students who are looking for work)
Others (including workers, office staff, graduated students who are looking for work)
Others (including workers, office staff, graduated students who are looking for work)
Others (including workers, office staff, graduated students who are looking for work)
Others (including workers, office staff, graduated students who are looking for work)
Others (including workers, office staff, graduated students who are looking for work)
Others (including workers, office staff, graduated students who are looking for work)
Student
Others (including workers, office staff, graduated students who are looking for work)
Others (including workers, office staff, graduated students who are looking for work)
Others (including workers, office staff, graduated students who are looking for work)
Others (including workers, office staff, graduated students who are looking for work)
Others (including workers, office staff, graduated students who are looking for work)
Others (including workers, office staff, graduated students who are looking for work)

Others (including workers, office staff, graduated students who are looking for work)
Others (including workers, office staff, graduated students who are looking for work)
Others (including workers, office staff, graduated students who are looking for work)
Others (including workers, office staff, graduated students who are looking for work)
Others (including workers, office staff, graduated students who are looking for work)
Others (including workers, office staff, graduated students who are looking for work)
Others (including workers, office staff, graduated students who are looking for work)
Others (including workers, office staff, graduated students who are looking for work)
Others (including workers, office staff, graduated students who are looking for work)
Others (including workers, office staff, graduated students who are looking for work)
Others (including workers, office staff, graduated students who are looking for work)
Others (including workers, office staff, graduated students who are looking for work)
Others (including workers, office staff, graduated students who are looking for work)
Others (including workers, office staff, graduated students who are looking for work)
Others (including workers, office staff, graduated students who are looking for work)
Others (including workers, office staff, graduated students who are looking for work)
Others (including workers, office staff, graduated students who are looking for work)
Others (including workers, office staff, graduated students who are looking for work)
Others (including workers, office staff, graduated students who are looking for work)
Others (including workers, office staff, graduated students who are looking for work)
Others (including workers, office staff, graduated students who are looking for work)
Others (including workers, office staff, graduated students who are looking for work)
Others (including workers, office staff, graduated students who are looking for work)
Student
Others (including workers, office staff, graduated students who are looking for work)
Others (including workers, office staff, graduated students who are looking for work)
Others (including workers, office staff, graduated students who are looking for work)
Others (including workers, office staff, graduated students who are looking for work)
Others (including workers, office staff, graduated students who are looking for work)
Others (including workers, office staff, graduated students who are looking for work)
Others (including workers, office staff, graduated students who are looking for work)
Others (including workers, office staff, graduated students who are looking for work)
Others (including workers, office staff, graduated students who are looking for work)
Others (including workers, office staff, graduated students who are looking for work)
Others (including workers, office staff, graduated students who are looking for work)
Others (including workers, office staff, graduated students who are looking for work)
Others (including workers, office staff, graduated students who are looking for work)
Others (including workers, office staff, graduated students who are looking for work)
Others (including workers, office staff, graduated students who are looking for work)
Others (including workers, office staff, graduated students who are looking for work)
Others (including workers, office staff, graduated students who are looking for work)
Others (including workers, office staff, graduated students who are looking for work)
Others (including workers, office staff, graduated students who are looking for work)
Others (including workers, office staff, graduated students who are looking for work)
Others (including workers, office staff, graduated students who are looking for work)
Others (including workers, office staff, graduated students who are looking for work)
Others (including workers, office staff, graduated students who are looking for work)
Others (including workers, office staff, graduated students who are looking for work)
Others (including workers, office staff, graduated students who are looking for work)
Student

Student
Others (including workers, office staff, graduated students who are looking for work)
Others (including workers, office staff, graduated students who are looking for work)
Others (including workers, office staff, graduated students who are looking for work)
Others (including workers, office staff, graduated students who are looking for work)
Others (including workers, office staff, graduated students who are looking for work)
Others (including workers, office staff, graduated students who are looking for work)
Others (including workers, office staff, graduated students who are looking for work)
Others (including workers, office staff, graduated students who are looking for work)
Others (including workers, office staff, graduated students who are looking for work)
Others (including workers, office staff, graduated students who are looking for work)
Others (including workers, office staff, graduated students who are looking for work)
Others (including workers, office staff, graduated students who are looking for work)
Others (including workers, office staff, graduated students who are looking for work)
Others (including workers, office staff, graduated students who are looking for work)
Others (including workers, office staff, graduated students who are looking for work)
Others (including workers, office staff, graduated students who are looking for work)
Others (including workers, office staff, graduated students who are looking for work)
Others (including workers, office staff, graduated students who are looking for work)
Others (including workers, office staff, graduated students who are looking for work)
Others (including workers, office staff, graduated students who are looking for work)
Others (including workers, office staff, graduated students who are looking for work)
Others (including workers, office staff, graduated students who are looking for work)
Others (including workers, office staff, graduated students who are looking for work)
Others (including workers, office staff, graduated students who are looking for work)
Others (including workers, office staff, graduated students who are looking for work)
Others (including workers, office staff, graduated students who are looking for work)
Others (including workers, office staff, graduated students who are looking for work)
Others (including workers, office staff, graduated students who are looking for work)
Others (including workers, office staff, graduated students who are looking for work)
Others (including workers, office staff, graduated students who are looking for work)
Others (including workers, office staff, graduated students who are looking for work)
Others (including workers, office staff, graduated students who are looking for work)
Others (including workers, office staff, graduated students who are looking for work)
Others (including workers, office staff, graduated students who are looking for work)
Others (including workers, office staff, graduated students who are looking for work)
Others (including workers, office staff, graduated students who are looking for work)
Others (including workers, office staff, graduated students who are looking for work)
Others (including workers, office staff, graduated students who are looking for work)
Others (including workers, office staff, graduated students who are looking for work)
Others (including workers, office staff, graduated students who are looking for work)
Others (including workers, office staff, graduated students who are looking for work)
Others (including workers, office staff, graduated students who are looking for work)
Others (including workers, office staff, graduated students who are looking for work)
Others (including workers, office staff, graduated students who are looking for work)
Others (including workers, office staff, graduated students who are looking for work)
Others (including workers, office staff, graduated students who are looking for work)
Others (including workers, office staff, graduated students who are looking for work)
Others (including workers, office staff, graduated students who are looking for work)
Others (including workers, office staff, graduated students who are looking for work)

Others (including workers, office staff, graduated students who are looking for work)
Others (including workers, office staff, graduated students who are looking for work)
Others (including workers, office staff, graduated students who are looking for work)
Others (including workers, office staff, graduated students who are looking for work)
Others (including workers, office staff, graduated students who are looking for work)
Others (including workers, office staff, graduated students who are looking for work)
Others (including workers, office staff, graduated students who are looking for work)
Others (including workers, office staff, graduated students who are looking for work)
Others (including workers, office staff, graduated students who are looking for work)
Others (including workers, office staff, graduated students who are looking for work)
Others (including workers, office staff, graduated students who are looking for work)
Others (including workers, office staff, graduated students who are looking for work)
Others (including workers, office staff, graduated students who are looking for work)
Others (including workers, office staff, graduated students who are looking for work)
Others (including workers, office staff, graduated students who are looking for work)
Others (including workers, office staff, graduated students who are looking for work)
Others (including workers, office staff, graduated students who are looking for work)
Others (including workers, office staff, graduated students who are looking for work)
Others (including workers, office staff, graduated students who are looking for work)
Others (including workers, office staff, graduated students who are looking for work)
Others (including workers, office staff, graduated students who are looking for work)
Others (including workers, office staff, graduated students who are looking for work)
Others (including workers, office staff, graduated students who are looking for work)
Others (including workers, office staff, graduated students who are looking for work)
Others (including workers, office staff, graduated students who are looking for work)
Others (including workers, office staff, graduated students who are looking for work)
Others (including workers, office staff, graduated students who are looking for work)
Others (including workers, office staff, graduated students who are looking for work)
Others (including workers, office staff, graduated students who are looking for work)
Others (including workers, office staff, graduated students who are looking for work)
Others (including workers, office staff, graduated students who are looking for work)
Others (including workers, office staff, graduated students who are looking for work)
Others (including workers, office staff, graduated students who are looking for work)
Others (including workers, office staff, graduated students who are looking for work)
Others (including workers, office staff, graduated students who are looking for work)
Others (including workers, office staff, graduated students who are looking for work)
Others (including workers, office staff, graduated students who are looking for work)
Others (including workers, office staff, graduated students who are looking for work)
Others (including workers, office staff, graduated students who are looking for work)
Others (including workers, office staff, graduated students who are looking for work)
Others (including workers, office staff, graduated students who are looking for work)
Others (including workers, office staff, graduated students who are looking for work)
Others (including workers, office staff, graduated students who are looking for work)
Others (including workers, office staff, graduated students who are looking for work)
Others (including workers, office staff, graduated students who are looking for work)
Others (including workers, office staff, graduated students who are looking for work)
Student
Others (including workers, office staff, graduated students who are looking for work)
Others (including workers, office staff, graduated students who are looking for work)
Others (including workers, office staff, graduated students who are looking for work)

Others (including workers, office staff, graduated students who are looking for work)
Others (including workers, office staff, graduated students who are looking for work)
Others (including workers, office staff, graduated students who are looking for work)
Others (including workers, office staff, graduated students who are looking for work)
Others (including workers, office staff, graduated students who are looking for work)
Others (including workers, office staff, graduated students who are looking for work)
Others (including workers, office staff, graduated students who are looking for work)
Others (including workers, office staff, graduated students who are looking for work)
Others (including workers, office staff, graduated students who are looking for work)
Others (including workers, office staff, graduated students who are looking for work)
Others (including workers, office staff, graduated students who are looking for work)
Others (including workers, office staff, graduated students who are looking for work)
Others (including workers, office staff, graduated students who are looking for work)
Others (including workers, office staff, graduated students who are looking for work)
Others (including workers, office staff, graduated students who are looking for work)
Others (including workers, office staff, graduated students who are looking for work)
Others (including workers, office staff, graduated students who are looking for work)
Others (including workers, office staff, graduated students who are looking for work)
Others (including workers, office staff, graduated students who are looking for work)
Others (including workers, office staff, graduated students who are looking for work)
Others (including workers, office staff, graduated students who are looking for work)
Others (including workers, office staff, graduated students who are looking for work)
Others (including workers, office staff, graduated students who are looking for work)
Others (including workers, office staff, graduated students who are looking for work)
Others (including workers, office staff, graduated students who are looking for work)
Others (including workers, office staff, graduated students who are looking for work)
Others (including workers, office staff, graduated students who are looking for work)
Others (including workers, office staff, graduated students who are looking for work)
Others (including workers, office staff, graduated students who are looking for work)
Others (including workers, office staff, graduated students who are looking for work)
Others (including workers, office staff, graduated students who are looking for work)
Others (including workers, office staff, graduated students who are looking for work)
Others (including workers, office staff, graduated students who are looking for work)
Others (including workers, office staff, graduated students who are looking for work)
Others (including workers, office staff, graduated students who are looking for work)
Others (including workers, office staff, graduated students who are looking for work)
Others (including workers, office staff, graduated students who are looking for work)
Others (including workers, office staff, graduated students who are looking for work)
Others (including workers, office staff, graduated students who are looking for work)
Others (including workers, office staff, graduated students who are looking for work)
Others (including workers, office staff, graduated students who are looking for work)
Others (including workers, office staff, graduated students who are looking for work)
Others (including workers, office staff, graduated students who are looking for work)
Others (including workers, office staff, graduated students who are looking for work)
Student
Others (including workers, office staff, graduated students who are looking for work)
Others (including workers, office staff, graduated students who are looking for work)
Others (including workers, office staff, graduated students who are looking for work)
Others (including workers, office staff, graduated students who are looking for work)
Others (including workers, office staff, graduated students who are looking for work)

Others (including workers, office staff, graduated students who are looking for work)
Others (including workers, office staff, graduated students who are looking for work)
Others (including workers, office staff, graduated students who are looking for work)
Others (including workers, office staff, graduated students who are looking for work)

7. Education level	8. Employment status	9. Motorcycle's brand name
Above high school	Part-time	Suzuki
Above high school	Full-time	Others
Above high school	Part-time	Honda
Above high school	Full-time	Others
Above high school		Suzuki
High school	Part-time	Honda
High school	Full-time	Others
Above high school	Full-time	Others
Above high school	Full-time	Yamaha
Above high school	Full-time	Yamaha
Above high school	Part-time	Suzuki
Above high school	Full-time	Others
Above high school	Full-time	Yamaha
Above high school	Part-time	Honda
Above high school	Full-time	Yamaha
Above high school	Full-time	Honda
High school		Suzuki
Above high school	Part-time	Suzuki
Above high school	Part-time	Others
Less than high school	Part-time	Yamaha
Above high school	Full-time	Honda
High school	Part-time	Honda
Less than high school	Full-time	Honda
High school	Part-time	Suzuki
Less than high school	Part-time	Suzuki
Above high school	Full-time	Others
High school	Full-time	Suzuki
High school	Full-time	Honda
High school	Part-time	Others
High school	Full-time	Yamaha
Less than high school	Part-time	Yamaha
Above high school	Part-time	Yamaha
Above high school	Full-time	Honda
High school	Part-time	Honda
Above high school	Part-time	Others
High school	Part-time	Honda
Above high school	Full-time	Honda
High school	Part-time	Honda
Above high school	Part-time	Yamaha
Above high school	Part-time	Yamaha
Above high school	Full-time	Yamaha
High school	Full-time	Honda
Above high school	Full-time	Yamaha
Above high school	Full-time	Yamaha
Above high school	Part-time	Others
Less than high school	Part-time	Others
Above high school	Part-time	Suzuki

Above high school	Full-time	Suzuki
Less than high school	Part-time	Others
Less than high school	Part-time	Others
Less than high school	Part-time	Others
Less than high school	Part-time	Yamaha
High school	Full-time	Suzuki
Above high school	Part-time	Yamaha
Above high school	Full-time	Honda
Above high school	Full-time	Honda
Above high school	Part-time	Others
Above high school	Part-time	Others
Above high school	Full-time	Suzuki
Above high school	Full-time	Honda
Above high school	Full-time	Suzuki
Above high school	Full-time	Honda
Above high school	Part-time	Others
Above high school	Part-time	Others
Above high school	Full-time	Others
Above high school	Full-time	Honda
Above high school	Full-time	Honda
High school	Full-time	Yamaha
High school	Part-time	Honda
Above high school	Part-time	Honda
Above high school	Part-time	Others
Above high school	Part-time	Others
Above high school	Full-time	Others
Less than high school	Part-time	Suzuki
Above high school	Part-time	Yamaha
High school	Full-time	Yamaha
Above high school	Part-time	Suzuki
Above high school	Part-time	Others
Above high school	Full-time	Honda
Above high school	Full-time	Others
Above high school	Part-time	Others
Above high school	Part-time	Honda
Above high school	Part-time	Yamaha
Above high school	Full-time	Honda
High school	Full-time	Others
Above high school	Part-time	Honda
High school	Part-time	Honda
High school	Full-time	Honda
Less than high school	Part-time	Others
Above high school	Part-time	
Above high school	Part-time	Yamaha
High school	Full-time	Honda
High school	Full-time	Honda
Less than high school	Part-time	Suzuki
Above high school	Part-time	Yamaha
Above high school	Full-time	Honda
Above high school	Part-time	Suzuki

Above high school	Part-time	Others
Less than high school	Full-time	Suzuki
Less than high school	Part-time	Honda
Above high school	Part-time	Yamaha
Above high school	Full-time	Others
High school	Full-time	Others
Above high school	Part-time	Suzuki
High school	Part-time	Others
High school	Part-time	Others
High school	Part-time	Others
Above high school	Part-time	Others
Above high school	Full-time	Yamaha
Above high school	Part-time	Yamaha
Above high school	Full-time	Honda
High school	Part-time	Others
High school	Full-time	Others
High school	Full-time	Others
High school	Part-time	Others
Above high school	Part-time	Yamaha
Above high school	Full-time	Others
Above high school	Full-time	Others
Above high school	Part-time	Honda
Above high school	Full-time	Yamaha
Less than high school	Full-time	Honda
Above high school	Full-time	Others
Above high school	Part-time	Yamaha
Above high school	Part-time	Yamaha
Less than high school	Part-time	Honda
Above high school	Part-time	Suzuki
Less than high school		Others
Above high school		Suzuki
Above high school	Part-time	Honda
Above high school	Full-time	Honda
Above high school	Part-time	Yamaha
Above high school	Full-time	Yamaha
High school	Part-time	Honda
High school	Part-time	Yamaha
High school	Part-time	Honda
Above high school	Full-time	Others
Above high school	Part-time	Others
Above high school	Full-time	Honda
Less than high school	Part-time	Yamaha
High school	Part-time	Yamaha
Above high school	Part-time	Others
Above high school	Full-time	Others
Above high school	Part-time	Others
Above high school	Part-time	Suzuki
Above high school	Full-time	Honda
Above high school		Others
Above high school	Full-time	Honda

Less than high school	Part-time	Yamaha
Above high school	Full-time	Honda
Above high school		Suzuki
Less than high school	Full-time	Honda
Less than high school	Part-time	Honda
Less than high school	Part-time	Honda
Less than high school	Full-time	Honda
Above high school	Full-time	Suzuki
High school	Full-time	Yamaha
Above high school	Part-time	Suzuki
Above high school	Part-time	Yamaha
Above high school	Full-time	Others
High school	Part-time	Others
Less than high school	Part-time	Others
Less than high school	Part-time	Others
Above high school	Full-time	Honda
Above high school	Part-time	Yamaha
Above high school	Part-time	Yamaha
Above high school	Full-time	Honda
Above high school	Full-time	Others
Above high school	Full-time	Honda
Above high school	Full-time	Honda
Above high school	Part-time	Others
Above high school	Part-time	Yamaha
Above high school	Full-time	Honda
Less than high school	Part-time	Yamaha
High school	Full-time	Others
High school	Full-time	Suzuki
Less than high school	Full-time	Yamaha
Above high school	Part-time	Honda
Above high school		Suzuki
Above high school		Yamaha
Less than high school	Full-time	Honda
Less than high school	Full-time	Honda
Above high school		Suzuki
Above high school	Full-time	Yamaha
Above high school	Full-time	Suzuki
Above high school	Full-time	Yamaha
Above high school	Part-time	Others
	Part-time	Yamaha
Above high school	Part-time	Others
Above high school	Full-time	Yamaha
Above high school	Part-time	Others
Above high school	Part-time	Others
High school	Part-time	Honda
Above high school	Part-time	Suzuki
High school	Full-time	Honda
Less than high school	Full-time	Yamaha
Above high school	Full-time	Others
Above high school	Part-time	Yamaha

Above high school	Full-time	Yamaha
Above high school	Full-time	Yamaha
High school	Full-time	Honda
Above high school	Full-time	Honda
Above high school	Full-time	Yamaha
Above high school	Part-time	Suzuki
Above high school	Part-time	Suzuki
Above high school	Part-time	Suzuki
Above high school	Full-time	Yamaha
High school	Full-time	Suzuki
Above high school	Part-time	Yamaha
Less than high school	Full-time	Others
Less than high school	Part-time	Suzuki
High school	Part-time	Suzuki
High school	Part-time	Others
Above high school	Part-time	Suzuki
Above high school	Part-time	Honda
Less than high school	Part-time	Suzuki
Less than high school	Part-time	Suzuki
Above high school	Part-time	Suzuki
Above high school	Part-time	Honda
Above high school	Part-time	Suzuki
Above high school	Part-time	Others
Above high school	Part-time	Others
Above high school	Part-time	Suzuki
Above high school	Part-time	Suzuki
Above high school	Part-time	Suzuki
Above high school	Part-time	Suzuki
Above high school	Part-time	Suzuki
Above high school	Part-time	Suzuki
Above high school	Part-time	Suzuki
Above high school	Part-time	Suzuki
Above high school	Part-time	Others
Above high school	Part-time	Others
Above high school	Part-time	Suzuki
High school	Part-time	Honda
Above high school	Part-time	Suzuki
Above high school	Part-time	Honda
Above high school	Part-time	Suzuki
Above high school	Part-time	Yamaha
High school	Part-time	Suzuki
Less than high school	Part-time	Suzuki
Above high school	Part-time	Suzuki
Above high school	Part-time	Suzuki
High school	Part-time	Suzuki
Above high school	Part-time	Suzuki
Above high school	Part-time	Suzuki
Above high school	Part-time	Suzuki
High school	Part-time	Suzuki
Above high school	Part-time	Suzuki

Above high school	Part-time	Suzuki
Above high school	Part-time	Suzuki
Above high school	Part-time	Honda
High school	Part-time	Others
High school	Part-time	Others
Above high school	Full-time	Others
Above high school	Part-time	Yamaha
Above high school	Part-time	Others
Above high school	Part-time	Suzuki
Above high school	Part-time	Honda
High school	Part-time	Suzuki
Above high school	Full-time	Honda
Above high school	Full-time	Others
High school	Part-time	Suzuki
High school	Full-time	Honda
Above high school	Full-time	Suzuki
High school	Part-time	Honda
High school	Full-time	Yamaha
Above high school	Full-time	Others
Above high school	Part-time	Others
Above high school	Full-time	Yamaha
Above high school	Part-time	Suzuki
Above high school	Part-time	Suzuki
Above high school	Full-time	Others
High school	Part-time	Suzuki
Above high school	Part-time	Suzuki
Above high school	Full-time	Others
Above high school	Part-time	Suzuki
Above high school	Part-time	Suzuki
Above high school	Part-time	Suzuki
Above high school	Full-time	Yamaha
Above high school	Full-time	Suzuki
Above high school	Part-time	Suzuki
Above high school	Full-time	Yamaha
Above high school	Part-time	Yamaha
Above high school	Full-time	Yamaha
Above high school	Part-time	Suzuki
Above high school	Part-time	Others
Above high school	Part-time	Suzuki
Above high school	Part-time	Suzuki
Above high school	Full-time	Yamaha
Above high school	Part-time	Suzuki
High school	Part-time	Suzuki
Above high school	Part-time	Suzuki
Above high school	Part-time	Suzuki
High school	Full-time	Others
Above high school	Part-time	Suzuki
Above high school	Part-time	Others
Above high school	Part-time	Suzuki
High school	Part-time	Suzuki

Above high school	Part-time	Others
High school	Part-time	Yamaha
Above high school	Full-time	Others
Above high school	Full-time	Honda
Above high school	Part-time	Honda
Above high school	Part-time	Suzuki
Above high school	Part-time	Suzuki
Above high school	Part-time	Suzuki
Less than high school	Part-time	Suzuki
Above high school	Part-time	Suzuki
Above high school	Part-time	Suzuki
Above high school	Part-time	Suzuki
Above high school	Part-time	Others
Above high school	Part-time	Others
Above high school	Part-time	Suzuki
High school	Part-time	Suzuki
Above high school	Part-time	Suzuki
Above high school	Part-time	Others
Above high school	Part-time	Suzuki
Above high school	Part-time	Suzuki
Above high school	Part-time	Yamaha
Above high school	Part-time	Suzuki
Above high school	Full-time	Yamaha
Above high school	Part-time	Honda
Above high school	Part-time	Others
Above high school	Full-time	Others
High school	Part-time	Others
Above high school	Full-time	Others
Above high school	Full-time	Suzuki
Above high school	Full-time	Honda
Above high school	Part-time	Suzuki
Above high school	Part-time	Suzuki
High school	Full-time	Others
Above high school	Part-time	Suzuki
High school	Part-time	Suzuki
Above high school	Part-time	Honda
Above high school	Part-time	Suzuki
Above high school	Part-time	Suzuki
High school	Part-time	Suzuki
Above high school	Full-time	Suzuki
Above high school	Part-time	Suzuki
Above high school	Part-time	Suzuki
Above high school	Part-time	Suzuki
Above high school	Full-time	Yamaha
Less than high school	Part-time	Suzuki
Above high school	Part-time	Honda
Above high school	Part-time	Suzuki
Above high school	Full-time	Suzuki
Above high school	Part-time	Honda
Above high school	Full-time	Yamaha

Above high school	Full-time	Suzuki
Above high school	Part-time	Yamaha
Above high school	Part-time	Suzuki
Above high school	Part-time	Suzuki
Above high school	Part-time	Suzuki
Above high school	Part-time	Others
Above high school	Part-time	Yamaha
Above high school	Part-time	Suzuki
High school	Full-time	Others
Above high school	Part-time	Others
Above high school	Full-time	Suzuki
Above high school	Part-time	Suzuki
Above high school	Part-time	Honda
Above high school	Part-time	Yamaha
Above high school	Part-time	Suzuki
High school	Part-time	Honda
Above high school	Part-time	Honda
Above high school	Part-time	Suzuki
High school	Part-time	Honda
High school	Part-time	Honda
Above high school	Part-time	Suzuki
Above high school	Part-time	Suzuki
Above high school	Part-time	Suzuki
Above high school	Full-time	Honda
Above high school	Part-time	Others
Above high school	Part-time	Honda
Above high school	Full-time	Suzuki
High school	Part-time	Suzuki
High school	Full-time	Yamaha
Above high school	Part-time	Yamaha
Above high school	Part-time	Suzuki
Above high school	Part-time	Suzuki
Above high school	Full-time	Suzuki
Above high school	Part-time	Others
Above high school	Full-time	Yamaha
Above high school	Full-time	Suzuki
Above high school	Full-time	Yamaha
Above high school	Full-time	Yamaha
Above high school	Full-time	Others
Above high school	Full-time	Others
Above high school	Part-time	Honda
Above high school	Part-time	Suzuki
High school	Part-time	Suzuki
Above high school	Part-time	Suzuki
Above high school	Part-time	Others
Above high school	Part-time	Suzuki
Above high school	Part-time	Others
Above high school	Part-time	Suzuki
Above high school	Part-time	Suzuki
Above high school	Part-time	Others

High school	Part-time	Honda
Above high school	Part-time	Suzuki
Above high school	Part-time	Others
Above high school	Part-time	Others

10. Is it registered motorcycle?	11. Do you have riding licence?
Yes	Yes
Yes	Yes
No	No
Yes	Yes
Yes	Yes
Yes	Yes
Yes	Yes
No	No
Yes	Yes
Yes	Yes
Yes	No
No	No
Yes	Yes
Yes	Yes
Yes	Yes
Yes	Yes
Yes	Yes
Yes	Yes
Yes	Yes
Yes	Yes
Yes	Yes
Yes	Yes
Yes	No
Yes	Yes
Yes	Yes
Yes	Yes
Yes	Yes
Yes	Yes
No	No
Yes	Yes
No	Yes
Yes	Yes
Yes	Yes
Yes	No
Yes	Yes
Yes	Yes
Yes	Yes
Yes	Yes
Yes	No
Yes	No
Yes	Yes
Yes	No
Yes	Yes
Yes	Yes
Yes	Yes
No	No
Yes	Yes

Yes	Yes
No	No
Yes	Yes
Yes	No
Yes	Yes
Yes	Yes
Yes	Yes
Yes	Yes
Yes	Yes
Yes	Yes
Yes	Yes
No	No
Yes	Yes
Yes	Yes
Yes	Yes
Yes	Yes
Yes	Yes
Yes	No
Yes	No
Yes	No
Yes	No
Yes	No
Yes	Yes
Yes	No
Yes	Yes
Yes	No
Yes	No
Yes	Yes
Yes	Yes
No	No
Yes	No
Yes	No
Yes	Yes
Yes	No
Yes	Yes
Yes	No
Yes	Yes
Yes	Yes
Yes	Yes
Yes	Yes
Yes	Yes
No	No
Yes	Yes
Yes	No
Yes	No
Yes	Yes
Yes	No
Yes	Yes
Yes	No

Yes	Yes
Yes	No
Yes	Yes
Yes	Yes
Yes	Yes
Yes	No
Yes	Yes
Yes	Yes
Yes	Yes
Yes	Yes
Yes	Yes
Yes	Yes
Yes	Yes
Yes	Yes
Yes	Yes
Yes	No
Yes	Yes
Yes	No
Yes	Yes
Yes	Yes
Yes	Yes
Yes	No
Yes	No
No	No
Yes	Yes
Yes	No
Yes	No
Yes	Yes
Yes	Yes
No	No
No	No
Yes	Yes
Yes	Yes
Yes	No
Yes	No
Yes	Yes
Yes	No
Yes	No
Yes	Yes
Yes	No
Yes	Yes
Yes	No
Yes	Yes
Yes	Yes
Yes	Yes
Yes	No
No	No
Yes	Yes
No	No
Yes	Yes

Yes	Yes
Yes	Yes
Yes	No
Yes	Yes
Yes	Yes
Yes	Yes
Yes	Yes
Yes	Yes
Yes	Yes
No	No
Yes	Yes
Yes	Yes
Yes	No
Yes	No
Yes	Yes
Yes	Yes
Yes	Yes
Yes	No
Yes	Yes
Yes	Yes
Yes	Yes
Yes	No
Yes	No
Yes	No
Yes	Yes
Yes	No
Yes	Yes
Yes	No
Yes	Yes
Yes	No
Yes	Yes
Yes	No
Yes	Yes
Yes	Yes
Yes	Yes
Yes	Yes
Yes	Yes
Yes	No
Yes	Yes
Yes	Yes
Yes	Yes
Yes	Yes
Yes	Yes
Yes	No
Yes	Yes
Yes	Yes
Yes	Yes
Yes	Yes
Yes	Yes
Yes	No

Yes	Yes
Yes	Yes
Yes	Yes
Yes	Yes
Yes	Yes
Yes	Yes
Yes	Yes
Yes	Yes
Yes	Yes
Yes	Yes
Yes	Yes
Yes	Yes
Yes	No
Yes	Yes
Yes	Yes
Yes	Yes
Yes	No
Yes	Yes
Yes	Yes
Yes	Yes
Yes	Yes
Yes	Yes
Yes	Yes
Yes	Yes
Yes	Yes
Yes	Yes
Yes	Yes
Yes	Yes
Yes	Yes
Yes	Yes
Yes	Yes
Yes	Yes
Yes	Yes
Yes	Yes
Yes	Yes
Yes	Yes
Yes	Yes
Yes	Yes
Yes	Yes
Yes	Yes
Yes	Yes
Yes	Yes
Yes	Yes
Yes	Yes
Yes	Yes
Yes	Yes
Yes	Yes
Yes	Yes
Yes	Yes
Yes	Yes

Yes	Yes
Yes	Yes
Yes	Yes
Yes	Yes
Yes	Yes
Yes	Yes
Yes	Yes
Yes	Yes
Yes	Yes
Yes	Yes
Yes	Yes
Yes	Yes
Yes	Yes
Yes	Yes
Yes	Yes
Yes	Yes
Yes	Yes
Yes	Yes
Yes	Yes
Yes	Yes
Yes	Yes
Yes	Yes
Yes	Yes
Yes	Yes
Yes	Yes
Yes	Yes
Yes	Yes
Yes	Yes
Yes	Yes
Yes	Yes
Yes	Yes
Yes	Yes
Yes	Yes
Yes	Yes
Yes	Yes
Yes	Yes
Yes	Yes
Yes	Yes
Yes	Yes
Yes	Yes
Yes	Yes
Yes	Yes
Yes	Yes
Yes	Yes
Yes	Yes
Yes	Yes
Yes	Yes
Yes	Yes
Yes	Yes
Yes	Yes

Yes	Yes
Yes	Yes
Yes	Yes
Yes	Yes
Yes	Yes
Yes	Yes
Yes	Yes
Yes	Yes
Yes	Yes
Yes	Yes
Yes	Yes
Yes	Yes
Yes	Yes
Yes	Yes
Yes	Yes
Yes	Yes
Yes	Yes
Yes	Yes
Yes	Yes
Yes	Yes
Yes	Yes
Yes	Yes
Yes	Yes
Yes	Yes
Yes	Yes
Yes	Yes
Yes	Yes
Yes	Yes
Yes	Yes
Yes	Yes
Yes	Yes
Yes	Yes
Yes	Yes
Yes	Yes
Yes	Yes
Yes	Yes
Yes	Yes
Yes	Yes
Yes	Yes
Yes	Yes
Yes	Yes
Yes	Yes
Yes	Yes
Yes	Yes
Yes	Yes
Yes	Yes
Yes	Yes
Yes	Yes
Yes	Yes
Yes	Yes

Yes	Yes
Yes	Yes
Yes	Yes
Yes	Yes
Yes	Yes
Yes	Yes
Yes	Yes
Yes	Yes
Yes	Yes
Yes	Yes
Yes	Yes
Yes	Yes
Yes	Yes
Yes	Yes
Yes	Yes
Yes	Yes
Yes	Yes
Yes	Yes
Yes	Yes
Yes	Yes
Yes	Yes
Yes	Yes
Yes	Yes
Yes	Yes
Yes	Yes
Yes	Yes
Yes	Yes
Yes	Yes
Yes	Yes
Yes	Yes
Yes	Yes
Yes	Yes
Yes	Yes
Yes	Yes
Yes	Yes
Yes	Yes
Yes	Yes
Yes	Yes
Yes	Yes
Yes	Yes
Yes	Yes
Yes	Yes
Yes	Yes
Yes	Yes
Yes	Yes
Yes	Yes
Yes	Yes
Yes	Yes
Yes	Yes
Yes	Yes

Yes	Yes
Yes	Yes
Yes	Yes
Yes	Yes

12. Motorcycle ownership	13. Daily travel distance
Inherited from relatives	<20 km
Bought with their own money	>=50 km
Inherited from relatives	<20 km
Bought with their own money	>=30 km & <40 km
Bought with their own money	>=50 km
Bought with their own money	>=50 km
Bought with their own money	>=50 km
Inherited from relatives	<20 km
Bought with their own money	<20 km
Bought with their own money	>=50 km
Inherited from relatives	<20 km
Inherited from relatives	>=50 km
Bought with their own money	<20 km
Inherited from relatives	<20 km
Bought with their own money	>=50 km
Bought with their own money	<20 km
Bought with their own money	>=30 km & <40 km
Bought with their own money	<20 km
Inherited from relatives	>=20 km & <30 km
Bought with their own money	>=20 km & <30 km
Bought with their own money	>=20 km & <30 km
Inherited from relatives	>=20 km & <30 km
Bought with their own money	>=30 km & <40 km
Inherited from relatives	>=20 km & <30 km
Bought with their own money	>=50 km
Inherited from relatives	>=20 km & <30 km
Inherited from relatives	>=20 km & <30 km
Inherited from relatives	>=30 km & <40 km
Inherited from relatives	>=50 km
Bought with their own money	>=30 km & <40 km
Bought with their own money	>=50 km
Inherited from relatives	>=20 km & <30 km
Bought with their own money	>=50 km
Inherited from relatives	>=20 km & <30 km
Inherited from relatives	>=30 km & <40 km
Bought with their own money	>=30 km & <40 km
Bought with their own money	>=30 km & <40 km
Inherited from relatives	>=30 km & <40 km
Inherited from relatives	>=20 km & <30 km
Bought with their own money	>=30 km & <40 km
Bought with their own money	>=30 km & <40 km
Bought with their own money	>=30 km & <40 km
Bought with their own money	>=50 km
Bought with their own money	>=50 km
Bought with their own money	>=50 km
Inherited from relatives	>=50 km

Bought with their own money	>=20 km & <30 km
Bought with their own money	>=30 km & <40 km
Inherited from relatives	>=30 km & <40 km
Inherited from relatives	>=30 km & <40 km
Inherited from relatives	<20 km
Inherited from relatives	>=30 km & <40 km
Bought with their own money	>=30 km & <40 km
Inherited from relatives	>=20 km & <30 km
Bought with their own money	>=30 km & <40 km
Inherited from relatives	>=30 km & <40 km
Inherited from relatives	>=20 km & <30 km
Bought with their own money	>=20 km & <30 km
Inherited from relatives	>=30 km & <40 km
Bought with their own money	>=30 km & <40 km
Bought with their own money	>=30 km & <40 km
Bought with their own money	>=30 km & <40 km
Bought with their own money	>=30 km & <40 km
Inherited from relatives	>=20 km & <30 km
Inherited from relatives	>=20 km & <30 km
Bought with their own money	>=30 km & <40 km
Bought with their own money	>=30 km & <40 km
Bought with their own money	>=30 km & <40 km
Inherited from relatives	>=30 km & <40 km
Inherited from relatives	>=20 km & <30 km
Inherited from relatives	>=30 km & <40 km
Inherited from relatives	<20 km
Inherited from relatives	>=30 km & <40 km
Inherited from relatives	>=30 km & <40 km
Bought with their own money	>=30 km & <40 km
Bought with their own money	>=20 km & <30 km
Bought with their own money	>=50 km
Inherited from relatives	>=20 km & <30 km
Inherited from relatives	>=20 km & <30 km
Bought with their own money	>=20 km & <30 km
Inherited from relatives	<20 km
Inherited from relatives	>=20 km & <30 km
Inherited from relatives	>=20 km & <30 km
Inherited from relatives	>=30 km & <40 km
Bought with their own money	>=20 km & <30 km
Inherited from relatives	>=20 km & <30 km
Inherited from relatives	>=20 km & <30 km
Inherited from relatives	>=20 km & <30 km
Inherited from relatives	<20 km

Bought with their own money	>=20 km & <30 km
Bought with their own money	>=30 km & <40 km
Bought with their own money	>=20 km & <30 km
Bought with their own money	>=30 km & <40 km
Inherited from relatives	>=20 km & <30 km
Inherited from relatives	>=20 km & <30 km

Inherited from relatives	>=20 km & <30 km
Inherited from relatives	>=20 km & <30 km
Bought with their own money	>=20 km & <30 km
Inherited from relatives	<20 km
Inherited from relatives	>=30 km & <40 km
Inherited from relatives	>=20 km & <30 km
Inherited from relatives	>=30 km & <40 km
Bought with their own money	>=20 km & <30 km
Inherited from relatives	>=20 km & <30 km
Inherited from relatives	>=20 km & <30 km
Inherited from relatives	>=30 km & <40 km
Inherited from relatives	>=20 km & <30 km
Bought with their own money	>=20 km & <30 km
Inherited from relatives	>=20 km & <30 km
Inherited from relatives	>=20 km & <30 km
Inherited from relatives	>=20 km & <30 km
Inherited from relatives	>=20 km & <30 km
Bought with their own money	>=30 km & <40 km
Inherited from relatives	>=20 km & <30 km
Bought with their own money	>=20 km & <30 km
Inherited from relatives	>=30 km & <40 km
Bought with their own money	>=50 km
Inherited from relatives	>=30 km & <40 km
Bought with their own money	>=50 km
Inherited from relatives	>=50 km
Bought with their own money	>=20 km & <30 km
Inherited from relatives	>=20 km & <30 km
Inherited from relatives	>=20 km & <30 km
Inherited from relatives	>=20 km & <30 km
Inherited from relatives	>=20 km & <30 km
Inherited from relatives	>=30 km & <40 km
Bought with their own money	>=50 km
Bought with their own money	>=50 km
Bought with their own money	>=20 km & <30 km
Inherited from relatives	>=20 km & <30 km
Inherited from relatives	>=20 km & <30 km
Inherited from relatives	>=20 km & <30 km
Inherited from relatives	>=30 km & <40 km
Bought with their own money	>=20 km & <30 km
Bought with their own money	>=20 km & <30 km
Bought with their own money	>=20 km & <30 km
Inherited from relatives	>=20 km & <30 km
Inherited from relatives	<20 km
Inherited from relatives	<20 km
Inherited from relatives	>=20 km & <30 km
Inherited from relatives	>=30 km & <40 km
Inherited from relatives	>=20 km & <30 km
Inherited from relatives	>=30 km & <40 km
Bought with their own money	>=50 km
Inherited from relatives	<20 km

Bought with their own money	>=50 km
Bought with their own money	>=50 km
Bought with their own money	>=50 km
Inherited from relatives	>=50 km
Bought with their own money	>=50 km
Bought with their own money	>=50 km
Inherited from relatives	<20 km
Bought with their own money	>=50 km
Bought with their own money	>=50 km
Bought with their own money	>=50 km
Inherited from relatives	>=20 km & <30 km
Bought with their own money	>=50 km
Inherited from relatives	>=30 km & <40 km
Bought with their own money	>=20 km & <30 km
Inherited from relatives	>=30 km & <40 km
Inherited from relatives	>=20 km & <30 km
Bought with their own money	>=20 km & <30 km
Bought with their own money	>=20 km & <30 km
Inherited from relatives	>=20 km & <30 km
Bought with their own money	>=20 km & <30 km
Bought with their own money	>=30 km & <40 km
Bought with their own money	>=20 km & <30 km
Bought with their own money	>=30 km & <40 km
Bought with their own money	>=20 km & <30 km
Inherited from relatives	>=20 km & <30 km
Bought with their own money	>=20 km & <30 km
Bought with their own money	>=20 km & <30 km
Bought with their own money	>=20 km & <30 km
Bought with their own money	>=20 km & <30 km
Bought with their own money	>=20 km & <30 km
Bought with their own money	>=20 km & <30 km
Bought with their own money	>=50 km
Bought with their own money	>=50 km
Bought with their own money	>=50 km
Bought with their own money	>=50 km
Bought with their own money	>=50 km
Bought with their own money	>=50 km
Bought with their own money	>=20 km & <30 km
Bought with their own money	>=20 km & <30 km
Bought with their own money	>=30 km & <40 km
Bought with their own money	>=20 km & <30 km
Inherited from relatives	>=20 km & <30 km
Inherited from relatives	>=20 km & <30 km
Bought with their own money	>=20 km & <30 km
Inherited from relatives	>=20 km & <30 km
Inherited from relatives	>=20 km & <30 km
Inherited from relatives	>=20 km & <30 km
Inherited from relatives	>=20 km & <30 km
Inherited from relatives	>=20 km & <30 km
Inherited from relatives	>=20 km & <30 km

Bought with their own money	>=30 km & <40 km
Bought with their own money	>=20 km & <30 km
Bought with their own money	>=20 km & <30 km
Bought with their own money	>=30 km & <40 km
Bought with their own money	>=20 km & <30 km
Inherited from relatives	>=20 km & <30 km
Inherited from relatives	>=20 km & <30 km
Bought with their own money	>=20 km & <30 km
Bought with their own money	>=20 km & <30 km
Bought with their own money	>=20 km & <30 km
Bought with their own money	>=30 km & <40 km
Inherited from relatives	>=30 km & <40 km
Inherited from relatives	>=30 km & <40 km
Bought with their own money	>=30 km & <40 km
Bought with their own money	>=20 km & <30 km
Bought with their own money	>=20 km & <30 km
Bought with their own money	>=30 km & <40 km
Bought with their own money	>=20 km & <30 km
Inherited from relatives	>=30 km & <40 km
Bought with their own money	>=20 km & <30 km
Bought with their own money	>=30 km & <40 km
Bought with their own money	>=30 km & <40 km
Bought with their own money	>=30 km & <40 km
Bought with their own money	>=30 km & <40 km
Bought with their own money	>=30 km & <40 km
Bought with their own money	>=30 km & <40 km
Bought with their own money	>=30 km & <40 km
Bought with their own money	>=30 km & <40 km
Bought with their own money	>=30 km & <40 km
Bought with their own money	>=30 km & <40 km
Bought with their own money	>=30 km & <40 km
Bought with their own money	>=30 km & <40 km
Bought with their own money	>=30 km & <40 km
Bought with their own money	>=30 km & <40 km
Bought with their own money	>=30 km & <40 km
Bought with their own money	>=30 km & <40 km
Bought with their own money	>=30 km & <40 km
Inherited from relatives	>=30 km & <40 km
Inherited from relatives	>=30 km & <40 km
Bought with their own money	>=20 km & <30 km
Bought with their own money	>=30 km & <40 km
Bought with their own money	>=30 km & <40 km
Bought with their own money	>=30 km & <40 km
Bought with their own money	>=30 km & <40 km
Bought with their own money	>=30 km & <40 km
Bought with their own money	>=30 km & <40 km
Bought with their own money	>=30 km & <40 km
Bought with their own money	>=30 km & <40 km
Bought with their own money	>=30 km & <40 km
Bought with their own money	>=30 km & <40 km

Bought with their own money	>=30 km & <40 km
Bought with their own money	>=30 km & <40 km
Bought with their own money	>=30 km & <40 km
Bought with their own money	>=30 km & <40 km
Inherited from relatives	>=30 km & <40 km
Bought with their own money	>=30 km & <40 km
Bought with their own money	>=30 km & <40 km
Bought with their own money	>=30 km & <40 km
Bought with their own money	>=30 km & <40 km
Inherited from relatives	>=30 km & <40 km
Bought with their own money	>=20 km & <30 km
Bought with their own money	>=30 km & <40 km
Bought with their own money	>=30 km & <40 km
Bought with their own money	>=30 km & <40 km
Bought with their own money	>=30 km & <40 km
Bought with their own money	>=30 km & <40 km
Bought with their own money	>=30 km & <40 km
Bought with their own money	>=30 km & <40 km
Inherited from relatives	>=20 km & <30 km
Bought with their own money	>=30 km & <40 km
Inherited from relatives	>=20 km & <30 km
Inherited from relatives	>=20 km & <30 km
Inherited from relatives	>=20 km & <30 km
Bought with their own money	>=30 km & <40 km
Bought with their own money	>=30 km & <40 km
Inherited from relatives	>=30 km & <40 km
Bought with their own money	>=30 km & <40 km
Bought with their own money	>=30 km & <40 km
Bought with their own money	>=30 km & <40 km
Bought with their own money	>=30 km & <40 km
Bought with their own money	>=20 km & <30 km
Bought with their own money	>=30 km & <40 km
Bought with their own money	>=20 km & <30 km
Bought with their own money	>=30 km & <40 km
Bought with their own money	>=30 km & <40 km
Bought with their own money	>=30 km & <40 km
Bought with their own money	>=20 km & <30 km
Bought with their own money	>=30 km & <40 km
Bought with their own money	>=30 km & <40 km
Bought with their own money	>=30 km & <40 km
Bought with their own money	>=20 km & <30 km
Bought with their own money	>=30 km & <40 km
Bought with their own money	>=30 km & <40 km
Bought with their own money	>=30 km & <40 km
Bought with their own money	>=30 km & <40 km
Bought with their own money	>=30 km & <40 km
Bought with their own money	>=30 km & <40 km
Bought with their own money	>=30 km & <40 km
Bought with their own money	>=30 km & <40 km
Bought with their own money	>=30 km & <40 km

Bought with their own money	>=30 km & <40 km
Bought with their own money	>=30 km & <40 km
Bought with their own money	>=30 km & <40 km
Bought with their own money	>=20 km & <30 km
Bought with their own money	>=30 km & <40 km
Bought with their own money	>=30 km & <40 km
Bought with their own money	>=30 km & <40 km
Bought with their own money	>=30 km & <40 km
Bought with their own money	>=30 km & <40 km
Bought with their own money	>=30 km & <40 km
Bought with their own money	>=30 km & <40 km
Bought with their own money	>=30 km & <40 km
Bought with their own money	>=30 km & <40 km
Inherited from relatives	>=30 km & <40 km
Inherited from relatives	>=20 km & <30 km
Bought with their own money	>=20 km & <30 km
Bought with their own money	>=30 km & <40 km
Bought with their own money	>=30 km & <40 km
Inherited from relatives	>=30 km & <40 km
Bought with their own money	>=30 km & <40 km
Bought with their own money	>=20 km & <30 km
Bought with their own money	>=30 km & <40 km
Bought with their own money	>=30 km & <40 km
Bought with their own money	>=20 km & <30 km
Bought with their own money	>=30 km & <40 km
Bought with their own money	>=30 km & <40 km
Bought with their own money	>=30 km & <40 km
Bought with their own money	>=30 km & <40 km
Bought with their own money	>=20 km & <30 km
Bought with their own money	>=20 km & <30 km
Bought with their own money	>=20 km & <30 km
Inherited from relatives	>=20 km & <30 km
Bought with their own money	>=30 km & <40 km
Bought with their own money	>=30 km & <40 km
Bought with their own money	>=30 km & <40 km
Bought with their own money	>=30 km & <40 km
Bought with their own money	>=30 km & <40 km
Bought with their own money	>=30 km & <40 km
Bought with their own money	>=20 km & <30 km
Bought with their own money	>=20 km & <30 km
Bought with their own money	>=30 km & <40 km
Bought with their own money	>=30 km & <40 km
Bought with their own money	>=30 km & <40 km
Bought with their own money	>=30 km & <40 km
Bought with their own money	>=20 km & <30 km
Bought with their own money	>=30 km & <40 km
Bought with their own money	>=20 km & <30 km
Bought with their own money	>=30 km & <40 km
Inherited from relatives	>=30 km & <40 km
Bought with their own money	>=30 km & <40 km

Bought with their own money	>=30 km & <40 km
Inherited from relatives	>=30 km & <40 km
Bought with their own money	>=30 km & <40 km
Bought with their own money	>=20 km & <30 km
Bought with their own money	>=30 km & <40 km
Bought with their own money	>=30 km & <40 km
Bought with their own money	>=30 km & <40 km
Inherited from relatives	>=30 km & <40 km
Bought with their own money	>=30 km & <40 km
Bought with their own money	>=20 km & <30 km
Inherited from relatives	>=30 km & <40 km
Bought with their own money	>=30 km & <40 km
Bought with their own money	>=30 km & <40 km
Inherited from relatives	>=20 km & <30 km
Inherited from relatives	>=20 km & <30 km
Bought with their own money	>=30 km & <40 km
Bought with their own money	>=30 km & <40 km
Inherited from relatives	>=30 km & <40 km
Bought with their own money	>=30 km & <40 km
Bought with their own money	>=30 km & <40 km
Bought with their own money	>=30 km & <40 km
Inherited from relatives	>=30 km & <40 km
Bought with their own money	>=30 km & <40 km
Bought with their own money	>=30 km & <40 km
Bought with their own money	>=30 km & <40 km
Bought with their own money	>=30 km & <40 km
Bought with their own money	>=30 km & <40 km
Bought with their own money	>=30 km & <40 km
Bought with their own money	>=30 km & <40 km
Bought with their own money	>=30 km & <40 km
Bought with their own money	>=30 km & <40 km
Bought with their own money	>=30 km & <40 km
Bought with their own money	>=20 km & <30 km
Bought with their own money	>=30 km & <40 km
Bought with their own money	>=30 km & <40 km
Bought with their own money	>=20 km & <30 km
Inherited from relatives	>=30 km & <40 km
Bought with their own money	>=20 km & <30 km
Inherited from relatives	>=30 km & <40 km
Bought with their own money	>=30 km & <40 km
Bought with their own money	>=30 km & <40 km
Bought with their own money	>=30 km & <40 km
Bought with their own money	>=30 km & <40 km
Bought with their own money	>=30 km & <40 km
Bought with their own money	>=30 km & <40 km
Bought with their own money	>=20 km & <30 km
Bought with their own money	>=30 km & <40 km
Inherited from relatives	>=30 km & <40 km
Bought with their own money	>=20 km & <30 km
Bought with their own money	>=20 km & <30 km

Bought with their own money	>=30 km & <40 km
Bought with their own money	>=20 km & <30 km
Bought with their own money	>=30 km & <40 km
Bought with their own money	>=30 km & <40 km
Inherited from relatives	>=20 km & <30 km

14. Weekly working hours	15. Have you faced any road traffic accident over the last one year period?
<40 hours	No
>=60 hours	No
>=60 hours	No
>=60 hours	Yes
<40 hours	Yes
<40 hours	Yes
<40 hours	No
>=60 hours	No
>=60 hours	Yes
>=60 hours	No
>=40 hours & <60 hours	Yes
>=60 hours	No
>=60 hours	Yes
<40 hours	No
<40 hours	No
<40 hours	No
	No
>=40 hours & <60 hours	No
>=40 hours & <60 hours	No
>=60 hours	Yes
>=60 hours	Yes
>=40 hours & <60 hours	No
>=60 hours	Yes
>=40 hours & <60 hours	No
>=60 hours	Yes
>=60 hours	Yes
>=60 hours	No
>=60 hours	No
>=60 hours	No
>=40 hours & <60 hours	Yes
>=60 hours	No
>=40 hours & <60 hours	No
>=60 hours	No
>=60 hours	No
>=60 hours	No
>=60 hours	No
>=60 hours	No
>=60 hours	No
>=40 hours & <60 hours	Yes
>=60 hours	No
>=60 hours	Yes
>=60 hours	No
>=60 hours	Yes
>=60 hours	No
>=60 hours	No
>=60 hours	Yes

>=40 hours & <60 hours	Yes
>=40 hours & <60 hours	No
>=60 hours	No
>=60 hours	Yes
>=60 hours	Yes
>=60 hours	No
>=40 hours & <60 hours	Yes
>=60 hours	No
>=40 hours & <60 hours	No
>=40 hours & <60 hours	No
>=40 hours & <60 hours	No
>=60 hours	No
>=60 hours	No
>=60 hours	Yes
>=60 hours	Yes
>=40 hours & <60 hours	No
>=60 hours	No
>=40 hours & <60 hours	No
>=60 hours	No
>=60 hours	Yes
>=40 hours & <60 hours	Yes
>=60 hours	No
>=60 hours	Yes
>=60 hours	No
>=60 hours	No
<40 hours	No
>=40 hours & <60 hours	No
>=40 hours & <60 hours	Yes
>=60 hours	Yes
	Yes
<40 hours	No
>=40 hours & <60 hours	No
>=60 hours	Yes
>=60 hours	Yes
>=40 hours & <60 hours	No
>=40 hours & <60 hours	No
>=40 hours & <60 hours	No
>=40 hours & <60 hours	Yes
>=60 hours	No
>=40 hours & <60 hours	Yes
<40 hours	Yes
>=40 hours & <60 hours	No
>=60 hours	No
>=60 hours	No
>=40 hours & <60 hours	Yes
>=40 hours & <60 hours	No
>=40 hours & <60 hours	No
>=40 hours & <60 hours	No
>=40 hours & <60 hours	No
>=60 hours	No

>=40 hours & <60 hours	No
>=60 hours	No
>=40 hours & <60 hours	Yes
<40 hours	Yes
>=60 hours	No
>=60 hours	No
>=40 hours & <60 hours	No
>=40 hours & <60 hours	Yes
<40 hours	Yes
>=60 hours	Yes
>=40 hours & <60 hours	No
>=60 hours	Yes
>=60 hours	No
>=40 hours & <60 hours	Yes
>=40 hours & <60 hours	No
>=40 hours & <60 hours	No
>=40 hours & <60 hours	No
>=40 hours & <60 hours	No
>=60 hours	No
>=40 hours & <60 hours	No
>=40 hours & <60 hours	Yes
>=60 hours	Yes
<40 hours	No
>=60 hours	No
>=60 hours	Yes
>=40 hours & <60 hours	No
>=40 hours & <60 hours	No
>=40 hours & <60 hours	Yes
>=60 hours	No
>=60 hours	Yes
>=60 hours	No
<40 hours	Yes
>=60 hours	No
>=40 hours & <60 hours	Yes
>=40 hours & <60 hours	No
>=60 hours	Yes
>=40 hours & <60 hours	No
>=40 hours & <60 hours	Yes
>=40 hours & <60 hours	Yes
>=40 hours & <60 hours	No
>=60 hours	No
>=40 hours & <60 hours	No
>=40 hours & <60 hours	Yes
>=40 hours & <60 hours	No
>=40 hours & <60 hours	No
>=40 hours & <60 hours	Yes
>=40 hours & <60 hours	No
>=60 hours	No
>=60 hours	Yes
	No

>=60 hours	Yes
>=60 hours	Yes
>=60 hours	Yes
	No
>=60 hours	No
>=60 hours	No
>=40 hours & <60 hours	No
>=60 hours	Yes
>=60 hours	No
>=60 hours	Yes
<40 hours	No
>=60 hours	No
>=40 hours & <60 hours	Yes
>=40 hours & <60 hours	No
>=40 hours & <60 hours	Yes
>=40 hours & <60 hours	No
>=40 hours & <60 hours	Yes
>=40 hours & <60 hours	No
>=40 hours & <60 hours	No
>=40 hours & <60 hours	Yes
>=40 hours & <60 hours	Yes
>=40 hours & <60 hours	Yes
>=40 hours & <60 hours	Yes
>=40 hours & <60 hours	No
>=40 hours & <60 hours	No
>=40 hours & <60 hours	Yes
>=40 hours & <60 hours	No
>=40 hours & <60 hours	Yes
>=40 hours & <60 hours	Yes
>=40 hours & <60 hours	Yes
>=40 hours & <60 hours	Yes
	No
	No
>=60 hours	No
>=60 hours	No
	No
>=60 hours	No
>=40 hours & <60 hours	No
>=40 hours & <60 hours	Yes
>=40 hours & <60 hours	No
>=40 hours & <60 hours	Yes
<40 hours	No
>=60 hours	Yes
>=40 hours & <60 hours	No
>=40 hours & <60 hours	No
>=40 hours & <60 hours	Yes
>=40 hours & <60 hours	Yes
>=40 hours & <60 hours	Yes
>=60 hours	Yes
>=40 hours & <60 hours	No

>=40 hours & <60 hours	No
<40 hours	No
<40 hours	No
>=40 hours & <60 hours	Yes
>=40 hours & <60 hours	No
>=60 hours	Yes
>=40 hours & <60 hours	Yes
>=40 hours & <60 hours	Yes
>=60 hours	Yes
>=40 hours & <60 hours	Yes
>=40 hours & <60 hours	Yes
>=40 hours & <60 hours	Yes
>=40 hours & <60 hours	Yes
>=40 hours & <60 hours	Yes
>=40 hours & <60 hours	Yes
>=40 hours & <60 hours	Yes
>=60 hours	Yes
>=40 hours & <60 hours	No
>=40 hours & <60 hours	Yes
>=40 hours & <60 hours	Yes
>=40 hours & <60 hours	Yes
>=40 hours & <60 hours	Yes
>=40 hours & <60 hours	Yes
>=40 hours & <60 hours	Yes
>=40 hours & <60 hours	Yes
>=40 hours & <60 hours	Yes
>=40 hours & <60 hours	Yes
>=40 hours & <60 hours	No
>=40 hours & <60 hours	Yes
>=40 hours & <60 hours	Yes
>=40 hours & <60 hours	Yes
>=40 hours & <60 hours	Yes
>=40 hours & <60 hours	Yes
>=40 hours & <60 hours	Yes
>=40 hours & <60 hours	Yes
>=40 hours & <60 hours	Yes
>=40 hours & <60 hours	Yes
>=40 hours & <60 hours	Yes
>=40 hours & <60 hours	Yes
>=40 hours & <60 hours	Yes
>=40 hours & <60 hours	Yes
>=40 hours & <60 hours	Yes
>=40 hours & <60 hours	Yes
>=40 hours & <60 hours	Yes
>=40 hours & <60 hours	Yes
>=40 hours & <60 hours	Yes
>=40 hours & <60 hours	Yes
>=40 hours & <60 hours	Yes
>=40 hours & <60 hours	Yes
>=40 hours & <60 hours	Yes

>=40 hours & <60 hours	Yes
>=40 hours & <60 hours	Yes
>=40 hours & <60 hours	Yes
>=40 hours & <60 hours	Yes
>=40 hours & <60 hours	Yes
>=40 hours & <60 hours	Yes
>=40 hours & <60 hours	Yes
>=40 hours & <60 hours	Yes
>=40 hours & <60 hours	Yes
>=40 hours & <60 hours	Yes
>=40 hours & <60 hours	Yes
>=40 hours & <60 hours	Yes
>=40 hours & <60 hours	Yes
>=40 hours & <60 hours	Yes
>=40 hours & <60 hours	Yes
>=40 hours & <60 hours	Yes
>=40 hours & <60 hours	Yes
>=40 hours & <60 hours	Yes
>=40 hours & <60 hours	Yes
>=40 hours & <60 hours	Yes
>=40 hours & <60 hours	Yes
>=40 hours & <60 hours	Yes
>=40 hours & <60 hours	Yes
>=40 hours & <60 hours	Yes
>=40 hours & <60 hours	Yes
>=40 hours & <60 hours	Yes
>=40 hours & <60 hours	Yes
>=40 hours & <60 hours	Yes
>=40 hours & <60 hours	Yes
>=40 hours & <60 hours	Yes
>=40 hours & <60 hours	Yes
>=40 hours & <60 hours	Yes
>=40 hours & <60 hours	Yes
>=40 hours & <60 hours	Yes
>=40 hours & <60 hours	Yes
>=40 hours & <60 hours	Yes
>=40 hours & <60 hours	Yes
>=40 hours & <60 hours	Yes
>=40 hours & <60 hours	Yes
>=40 hours & <60 hours	Yes
>=40 hours & <60 hours	Yes
>=40 hours & <60 hours	Yes
>=40 hours & <60 hours	Yes
>=40 hours & <60 hours	Yes
>=40 hours & <60 hours	Yes
>=40 hours & <60 hours	Yes
>=40 hours & <60 hours	Yes
>=40 hours & <60 hours	Yes
>=40 hours & <60 hours	Yes
>=40 hours & <60 hours	Yes

>=40 hours & <60 hours	Yes
>=40 hours & <60 hours	Yes
>=40 hours & <60 hours	Yes
>=40 hours & <60 hours	Yes
>=40 hours & <60 hours	Yes
>=40 hours & <60 hours	Yes
>=40 hours & <60 hours	Yes
>=40 hours & <60 hours	Yes
>=40 hours & <60 hours	Yes
>=40 hours & <60 hours	Yes
>=40 hours & <60 hours	Yes
>=40 hours & <60 hours	Yes
>=40 hours & <60 hours	Yes
>=60 hours	Yes
>=40 hours & <60 hours	Yes
>=40 hours & <60 hours	Yes
>=40 hours & <60 hours	Yes
>=40 hours & <60 hours	Yes
>=40 hours & <60 hours	Yes
>=40 hours & <60 hours	Yes
>=40 hours & <60 hours	Yes
>=40 hours & <60 hours	Yes
>=40 hours & <60 hours	Yes
>=40 hours & <60 hours	Yes
>=40 hours & <60 hours	Yes
>=40 hours & <60 hours	Yes
>=40 hours & <60 hours	Yes
>=40 hours & <60 hours	Yes
>=40 hours & <60 hours	Yes
>=40 hours & <60 hours	Yes
>=40 hours & <60 hours	Yes
>=40 hours & <60 hours	Yes
>=40 hours & <60 hours	Yes
>=40 hours & <60 hours	Yes
>=40 hours & <60 hours	Yes
>=40 hours & <60 hours	Yes
>=40 hours & <60 hours	Yes
>=40 hours & <60 hours	Yes
>=40 hours & <60 hours	Yes
>=40 hours & <60 hours	Yes
>=40 hours & <60 hours	Yes
>=40 hours & <60 hours	Yes
>=40 hours & <60 hours	Yes
>=40 hours & <60 hours	Yes
>=40 hours & <60 hours	Yes
>=40 hours & <60 hours	Yes
>=40 hours & <60 hours	Yes
>=40 hours & <60 hours	Yes
>=40 hours & <60 hours	Yes
>=40 hours & <60 hours	Yes

>=40 hours & <60 hours	Yes
>=40 hours & <60 hours	Yes
>=40 hours & <60 hours	Yes
>=40 hours & <60 hours	Yes
>=40 hours & <60 hours	Yes
>=40 hours & <60 hours	Yes
>=40 hours & <60 hours	Yes
>=40 hours & <60 hours	Yes
>=40 hours & <60 hours	Yes
	Yes
>=40 hours & <60 hours	Yes
>=40 hours & <60 hours	Yes
>=40 hours & <60 hours	Yes
>=40 hours & <60 hours	Yes
>=40 hours & <60 hours	Yes
>=40 hours & <60 hours	Yes
>=40 hours & <60 hours	Yes
>=40 hours & <60 hours	Yes
>=40 hours & <60 hours	Yes
>=40 hours & <60 hours	Yes
>=40 hours & <60 hours	Yes
>=40 hours & <60 hours	Yes
>=40 hours & <60 hours	Yes
>=40 hours & <60 hours	Yes
>=40 hours & <60 hours	Yes
>=40 hours & <60 hours	Yes
>=40 hours & <60 hours	Yes
>=40 hours & <60 hours	Yes
>=40 hours & <60 hours	Yes
>=40 hours & <60 hours	Yes
>=40 hours & <60 hours	Yes
>=40 hours & <60 hours	Yes
>=40 hours & <60 hours	Yes
>=40 hours & <60 hours	Yes
<40 hours	Yes
>=40 hours & <60 hours	Yes
>=40 hours & <60 hours	Yes
>=40 hours & <60 hours	Yes
>=40 hours & <60 hours	Yes
>=40 hours & <60 hours	Yes
>=40 hours & <60 hours	Yes
>=40 hours & <60 hours	Yes
>=40 hours & <60 hours	Yes
>=40 hours & <60 hours	Yes
>=40 hours & <60 hours	Yes
>=40 hours & <60 hours	Yes
>=40 hours & <60 hours	Yes
>=40 hours & <60 hours	Yes
>=40 hours & <60 hours	Yes
>=40 hours & <60 hours	Yes

>=40 hours & <60 hours	Yes
>=40 hours & <60 hours	Yes
>=40 hours & <60 hours	Yes
>=40 hours & <60 hours	Yes
>=40 hours & <60 hours	Yes

16. Any injury caused by motorcycle road traffic accidents over the last year?

Yes

No
No
Yes
No
Yes
No
Yes
Yes
No
Yes
No
Yes
Yes
Yes

No
No
No
No
Yes

No
Yes
No
Yes
Yes
No
No
No
Yes
Yes
No
No
No

No

Yes

Yes

Yes
No
No
Yes

No
No
No
Yes
Yes
No
Yes
No
No
No
No
No
No
Yes
Yes
No
No
No
No
Yes
Yes
No
Yes
No
No
No
No
No
Yes
No
No
No
Yes
Yes
No
No
No
Yes
No
Yes
Yes
No
No
No
Yes
No
Yes
No
No
No

No
No
Yes
Yes
No
No
No
Yes
Yes
Yes
No
Yes
No
Yes
No
No
No
No
No
No
Yes
No
No
Yes
No
No
No
Yes
No
Yes
No
Yes
No
Yes
No
Yes
No
Yes
Yes
No
No
No
Yes
No
No
Yes
No
No
Yes
Yes

Yes
Yes
Yes
No
Yes
Yes
No
Yes
No
Yes
No
No
Yes
No
Yes
No
Yes
No
No
Yes
Yes
Yes
Yes
No
No
Yes
No
Yes
Yes
Yes
Yes
Yes
No
Yes
Yes
Yes
Yes
No
Yes
No
Yes
No
Yes
No
No
Yes
Yes
Yes
Yes
No

No
No
No
Yes
No
Yes
Yes
Yes
Yes
Yes
Yes
Yes
Yes
Yes
Yes
Yes
Yes
No
Yes
Yes
Yes
Yes
Yes
Yes
Yes
Yes
Yes
No
Yes
Yes
Yes
Yes
Yes
Yes
Yes
Yes
Yes
Yes
Yes
Yes
Yes
Yes
Yes
Yes
Yes
Yes
Yes
Yes
Yes
Yes

Yes
Yes
Yes
Yes
Yes
Yes
Yes
Yes
Yes
Yes
Yes
Yes
Yes
Yes
Yes
Yes
Yes
Yes
Yes
Yes
Yes
Yes
Yes
Yes
Yes
Yes
Yes
Yes
Yes
Yes
Yes
Yes
Yes
Yes
Yes
Yes
Yes
Yes
Yes
Yes
Yes
Yes
Yes
Yes
Yes
Yes
Yes
Yes
Yes
Yes

Yes
Yes
Yes
Yes
Yes
Yes
Yes
Yes
Yes
Yes
Yes
Yes
Yes
Yes
Yes
Yes
Yes
Yes
Yes
Yes
Yes
Yes
Yes
Yes
Yes
Yes
Yes
Yes
Yes
Yes
Yes
Yes
Yes
Yes
Yes
Yes
Yes
Yes
Yes
Yes
Yes
Yes
Yes
Yes
Yes
Yes
Yes
Yes
Yes
Yes

Yes
Yes
Yes
Yes
Yes
Yes
Yes
Yes
Yes
Yes
Yes
Yes
Yes
Yes
Yes
Yes
Yes
Yes
Yes
Yes
Yes
Yes
Yes
Yes
Yes
Yes
Yes
Yes
Yes
Yes
Yes
Yes
Yes
Yes
Yes
Yes
Yes
Yes
Yes
Yes
Yes
Yes
Yes
Yes
Yes
Yes
Yes
Yes
Yes
Yes

Yes
Yes
Yes
Yes
Yes

17. Accident severity types that you faced over the last one year period

Moderate

Minor
Minor

Moderate

Moderate
Severe

Moderate

Moderate
Severe
Minor

Minor

Minor
Moderate

Minor

Minor
Moderate

Minor
Severe
Minor

Minor

Moderate

Moderate

Minor
Minor

Moderate

Minor

Minor
Moderate

Minor

Minor
Moderate
Severe

Moderate
Severe

Moderate

Minor

Moderate
Moderate
Moderate
Minor

Moderate
Moderate

Severe

Minor
Moderate

Moderate

Moderate

Severe
Minor

Moderate
Minor
Moderate

Minor
Moderate
Minor

Moderate

Moderate
Minor

Minor
Minor

Moderate

Severe

Moderate

Minor

Moderate

Severe
Minor

Minor

Severe

Moderate
Minor

Moderate
Minor
Minor

Minor
Minor
Minor
Minor

Moderate

Minor

Minor

Severe

Moderate
Minor
Moderate
Severe

Moderate

Minor
Severe
Minor
Moderate
Minor

Minor
Moderate
Minor
Minor

Minor

Moderate

Moderate

Minor
Minor
Moderate
Severe

Minor

Moderate
Moderate
Moderate
Moderate
Minor
Moderate
Minor
Minor
Moderate
Moderate
Minor
Moderate

Moderate
Moderate
Minor
Minor
Moderate
Minor
Minor
Moderate
Moderate

Moderate
Moderate
Severe
Moderate
Moderate
Minor
Minor
Minor
Moderate
Minor
Moderate
Minor
Moderate
Moderate
Moderate
Minor
Moderate
Minor
Moderate
Moderate
Minor
Moderate

Minor
Minor
Moderate
Moderate
Moderate
Moderate
Moderate
Moderate
Minor
Minor
Minor
Moderate
Moderate
Moderate
Moderate
Moderate
Moderate
Moderate
Moderate
Moderate
Moderate
Moderate
Minor
Moderate
Moderate
Moderate
Moderate
Moderate
Moderate
Minor
Minor
Minor
Moderate
Moderate
Moderate
Moderate
Moderate
Moderate
Moderate
Minor
Moderate
Moderate
Moderate
Minor
Moderate
Minor
Moderate
Minor
Minor
Moderate

Moderate
Moderate
Moderate
Moderate
Moderate
Moderate
Moderate
Moderate
Minor
Minor
Minor
Minor
Minor
Moderate
Moderate
Minor
Moderate
Moderate
Moderate
Moderate
Moderate
Moderate
Moderate
Moderate
Moderate
Moderate
Moderate
Moderate
Moderate
Moderate
Moderate
Moderate
Moderate
Minor
Moderate
Moderate
Moderate
Severe
Moderate
Moderate
Moderate
Moderate
Moderate
Minor
Moderate
Severe
Moderate
Minor
Minor
Moderate

Minor
Moderate
Moderate
Minor
Severe
Minor
Moderate
Moderate
Minor
Minor
Moderate
Moderate
Minor
Moderate
Moderate
Severe
Moderate
Moderate
Minor
Moderate
Moderate
Moderate
Moderate
Minor
Severe
Moderate
Moderate
Moderate
Moderate
Moderate
Moderate
Severe
Moderate
Moderate
Moderate
Moderate
Moderate
Moderate
Moderate
Moderate
Moderate
Moderate
Moderate
Minor
Moderate
Minor
Minor
Moderate
Minor
Minor

Moderate
Moderate
Severe
Minor
Moderate

18. Weather condition while accident took place

Rainy

Rainy
Rainy
Misty
Sunny

Others
Sunny

Sunny

Rainy
Rainy
Others

Others
Sunny

Rainy
Others
Rainy
Sunny

Rainy
Misty

Others
Rainy
Rainy

Others

Misty

Rainy

Rainy
Others

Others

Rainy

Rainy
Others

Rainy

Sunny
Sunny
Rainy

Rainy
Misty

Misty

Others

Rainy
Rainy
Rainy
Misty

Sunny
Rainy

Rainy

Misty
Sunny

Others
Rainy

Sunny

Rainy
Sunny

Rainy
Others
Others

Misty
Misty
Misty

Others

Others
Others

Rainy
Others

Others

Sunny

Others

Sunny

Sunny

Rainy
Rainy

Others

Misty

Rainy
Others

Others
Others
Others

Others
Rainy
Others
Others

Others

Rainy

Others

Rainy

Others
Misty
Others
Rainy

Misty

Others
Rainy
Rainy
Rainy
Others

Others
Misty
Others
Others

Sunny

Others

Sunny

Misty
Others
Sunny
Sunny

Misty

Rainy
Others
Rainy
Misty
Misty
Rainy
Others
Others
Sunny
Sunny
Sunny
Misty

Others
Rainy
Others
Sunny
Others
Misty
Rainy
Sunny
Others

Sunny
Others
Sunny
Others
Others
Others
Misty
Others
Sunny
Rainy
Rainy
Rainy
Sunny
Sunny
Misty
Rainy
Sunny
Rainy
Rainy
Rainy
Others
Sunny

Others
Others
Sunny
Others
Rainy
Others
Misty
Rainy
Rainy
Sunny
Rainy
Misty
Rainy
Rainy
Rainy
Rainy
Rainy
Rainy
Rainy
Others
Others
Rainy
Others
Others
Misty
Rainy
Others
Others
Misty
Others
Sunny
Rainy
Misty
Sunny
Rainy
Misty
Rainy
Misty
Rainy
Sunny
Rainy
Rainy
Sunny
Others
Others
Others
Others
Rainy
Others
Sunny

Rainy
Rainy
Rainy
Rainy
Rainy
Rainy
Misty
Misty
Misty
Sunny
Misty
Sunny
Misty
Rainy
Rainy
Rainy
Misty
Misty
Misty
Misty
Misty
Sunny
Others
Misty
Rainy
Rainy
Rainy
Misty
Misty
Rainy
Rainy
Others
Misty
Rainy
Sunny
Misty
Rainy
Rainy
Sunny
Rainy
Rainy
Others
Misty
Others
Rainy
Sunny
Rainy
Others
Rainy
Rainy

Rainy
Rainy
Misty
Misty
Sunny
Rainy
Sunny
Rainy
Others
Sunny
Rainy
Rainy
Sunny
Misty
Misty
Rainy
Rainy
Others
Misty
Rainy
Misty
Rainy
Sunny
Rainy
Rainy
Rainy
Rainy
Rainy
Misty
Rainy
Misty
Misty
Rainy
Sunny
Sunny
Rainy
Rainy
Rainy
Misty
Misty
Rainy
Rainy
Rainy
Others
Others
Others
Sunny
Sunny
Misty
Misty

Rainy
Rainy
Sunny
Others
Misty

19. Do you talk on the phone while riding motorcycle? If yes, how often?	20. Drinking status
Often (few times a week)	Sometimes
Sometimes (few times a mouth)	Never
Sometimes (few times a mouth)	Never
Sometimes (few times a mouth)	Never
	Never
Sometimes (few times a mouth)	Never
Sometimes (few times a mouth)	Never
Sometimes (few times a mouth)	Never
Sometimes (few times a mouth)	Never
Sometimes (few times a mouth)	Never
Sometimes (few times a mouth)	Never
Sometimes (few times a mouth)	Sometimes
Sometimes (few times a mouth)	Sometimes
	Never
Sometimes (few times a mouth)	Never
Sometimes (few times a mouth)	Sometimes
Sometimes (few times a mouth)	Never
Sometimes (few times a mouth)	Sometimes
Often (few times a week)	Never
Often (few times a week)	Sometimes
	Never
Often (few times a week)	Sometimes
Sometimes (few times a mouth)	Sometimes
Regularly (few times a day)	Sometimes
Sometimes (few times a mouth)	Never
Sometimes (few times a mouth)	Sometimes
Sometimes (few times a mouth)	Never
Sometimes (few times a mouth)	Never
Sometimes (few times a mouth)	Never
Sometimes (few times a mouth)	Sometimes
Sometimes (few times a mouth)	Sometimes
Sometimes (few times a mouth)	Never
Sometimes (few times a mouth)	Never
Sometimes (few times a mouth)	Never
Sometimes (few times a mouth)	Sometimes
Sometimes (few times a mouth)	Sometimes
Sometimes (few times a mouth)	Never
Sometimes (few times a mouth)	Never
Sometimes (few times a mouth)	Sometimes
Often (few times a week)	Never
Sometimes (few times a mouth)	Never
Often (few times a week)	Sometimes
Sometimes (few times a mouth)	Never
Sometimes (few times a mouth)	Never
Sometimes (few times a mouth)	Sometimes

Sometimes (few times a mouth)	Never
Sometimes (few times a mouth)	Never
	Never
Sometimes (few times a mouth)	Never
Sometimes (few times a mouth)	Never
Sometimes (few times a mouth)	Sometimes
Sometimes (few times a mouth)	Never
Sometimes (few times a mouth)	Sometimes
Sometimes (few times a mouth)	Sometimes
Often (few times a week)	Sometimes
Sometimes (few times a mouth)	Sometimes
Sometimes (few times a mouth)	Sometimes
Sometimes (few times a mouth)	Never
Sometimes (few times a mouth)	Never
Sometimes (few times a mouth)	Never
Often (few times a week)	Sometimes
Often (few times a week)	Never
Sometimes (few times a mouth)	Sometimes
Sometimes (few times a mouth)	Sometimes
Sometimes (few times a mouth)	Sometimes
Often (few times a week)	Never
Sometimes (few times a mouth)	Sometimes
Sometimes (few times a mouth)	Never
Sometimes (few times a mouth)	Never
Sometimes (few times a mouth)	Never
Sometimes (few times a mouth)	Never
Sometimes (few times a mouth)	Sometimes
Sometimes (few times a mouth)	Sometimes
Sometimes (few times a mouth)	Never
Sometimes (few times a mouth)	Never
Sometimes (few times a mouth)	Never
Sometimes (few times a mouth)	Never
Often (few times a week)	Never
Often (few times a week)	Never
Sometimes (few times a mouth)	Never
Sometimes (few times a mouth)	Never
Sometimes (few times a mouth)	Never
Sometimes (few times a mouth)	Never
Sometimes (few times a mouth)	Sometimes
Sometimes (few times a mouth)	Never
Sometimes (few times a mouth)	Never
Sometimes (few times a mouth)	Never
Sometimes (few times a mouth)	Sometimes
	Never
Sometimes (few times a mouth)	Never
Sometimes (few times a mouth)	Sometimes
Sometimes (few times a mouth)	Sometimes
Often (few times a week)	Sometimes
Sometimes (few times a mouth)	Never
Sometimes (few times a mouth)	Never

Sometimes (few times a mouth)	Never
Sometimes (few times a mouth)	Never
Often (few times a week)	Sometimes
Often (few times a week)	Sometimes
Sometimes (few times a mouth)	Never
Often (few times a week)	Never
Often (few times a week)	Never
Sometimes (few times a mouth)	Sometimes
Sometimes (few times a mouth)	Sometimes
Sometimes (few times a mouth)	Never
Often (few times a week)	Never
Sometimes (few times a mouth)	Never
Often (few times a week)	Never
Often (few times a week)	Sometimes
Sometimes (few times a mouth)	Never
Sometimes (few times a mouth)	Sometimes
Sometimes (few times a mouth)	Never
Sometimes (few times a mouth)	Never
Sometimes (few times a mouth)	Sometimes
Sometimes (few times a mouth)	Never
Often (few times a week)	Sometimes
Sometimes (few times a mouth)	Sometimes
Sometimes (few times a mouth)	Never
Sometimes (few times a mouth)	Never
	Never
Sometimes (few times a mouth)	Never
Sometimes (few times a mouth)	Never
Sometimes (few times a mouth)	Never
Sometimes (few times a mouth)	Sometimes
Sometimes (few times a mouth)	Never
Sometimes (few times a mouth)	Sometimes
Sometimes (few times a mouth)	Sometimes
Sometimes (few times a mouth)	Never
Sometimes (few times a mouth)	Never
Sometimes (few times a mouth)	Never
Sometimes (few times a mouth)	Sometimes
Sometimes (few times a mouth)	Never
Sometimes (few times a mouth)	Never
Sometimes (few times a mouth)	Never
Sometimes (few times a mouth)	Never
Sometimes (few times a mouth)	Sometimes
Sometimes (few times a mouth)	Never
Sometimes (few times a mouth)	Never
	Never
Sometimes (few times a mouth)	Never
Sometimes (few times a mouth)	Sometimes
Often (few times a week)	Never
Sometimes (few times a mouth)	Never
Sometimes (few times a mouth)	Sometimes
Sometimes (few times a mouth)	Never

	Never
Sometimes (few times a mouth)	Sometimes
	Never
	Never
Sometimes (few times a mouth)	Never
	Never
Sometimes (few times a mouth)	Never
	Never
	Never
Sometimes (few times a mouth)	Never
Sometimes (few times a mouth)	Sometimes
Sometimes (few times a mouth)	Never
Sometimes (few times a mouth)	Sometimes
Sometimes (few times a mouth)	Never
Sometimes (few times a mouth)	Never
Sometimes (few times a mouth)	Never
Sometimes (few times a mouth)	Sometimes
Often (few times a week)	Never
Sometimes (few times a mouth)	Never
Sometimes (few times a mouth)	Sometimes
Sometimes (few times a mouth)	Never
Often (few times a week)	Never
Sometimes (few times a mouth)	Sometimes
Sometimes (few times a mouth)	Never
Sometimes (few times a mouth)	Never
Sometimes (few times a mouth)	Never
Sometimes (few times a mouth)	Never
Sometimes (few times a mouth)	Sometimes
Sometimes (few times a mouth)	Sometimes
Sometimes (few times a mouth)	Never
Sometimes (few times a mouth)	Sometimes
Sometimes (few times a mouth)	Never
Sometimes (few times a mouth)	Never
Sometimes (few times a mouth)	Never
Sometimes (few times a mouth)	Never
	Never
Sometimes (few times a mouth)	Never
Sometimes (few times a mouth)	Never
Sometimes (few times a mouth)	Sometimes
Sometimes (few times a mouth)	Never
Sometimes (few times a mouth)	Sometimes
Sometimes (few times a mouth)	Never
Sometimes (few times a mouth)	Sometimes
Sometimes (few times a mouth)	Never
Sometimes (few times a mouth)	Never
Sometimes (few times a mouth)	Never
Sometimes (few times a mouth)	Sometimes
Often (few times a week)	Never
Sometimes (few times a mouth)	Sometimes
Sometimes (few times a mouth)	Never

Sometimes (few times a mouth)	Never
Sometimes (few times a mouth)	Never
Sometimes (few times a mouth)	Never
Sometimes (few times a mouth)	Never
Sometimes (few times a mouth)	Sometimes
Sometimes (few times a mouth)	Never
Sometimes (few times a mouth)	Sometimes
Sometimes (few times a mouth)	Never
Sometimes (few times a mouth)	Never
Often (few times a week)	Never
Often (few times a week)	Never
Sometimes (few times a mouth)	Never
Sometimes (few times a mouth)	Never
Sometimes (few times a mouth)	Never
Often (few times a week)	Sometimes
Often (few times a week)	Never
Often (few times a week)	Sometimes
Often (few times a week)	Sometimes
Often (few times a week)	Never
Often (few times a week)	Never
Sometimes (few times a mouth)	Sometimes
Sometimes (few times a mouth)	Never
Sometimes (few times a mouth)	Never
Sometimes (few times a mouth)	Never
Often (few times a week)	Never
Often (few times a week)	Never
Often (few times a week)	Never
Often (few times a week)	Never
Often (few times a week)	Never
Often (few times a week)	Sometimes
Often (few times a week)	Never
Often (few times a week)	Never
Often (few times a week)	Never
Often (few times a week)	Never
Often (few times a week)	Never
Sometimes (few times a mouth)	Never
Sometimes (few times a mouth)	Never
Often (few times a week)	Sometimes
Often (few times a week)	Never
Sometimes (few times a mouth)	Never
Often (few times a week)	Sometimes
Often (few times a week)	Never
Often (few times a week)	Never
Often (few times a week)	Never
Often (few times a week)	Never
Often (few times a week)	Never
Sometimes (few times a mouth)	Never
Often (few times a week)	Sometimes
Often (few times a week)	Never
Often (few times a week)	Never

Sometimes (few times a mouth)	Never
Sometimes (few times a mouth)	Never
Often (few times a week)	Never
Sometimes (few times a mouth)	Never
Often (few times a week)	Never
Sometimes (few times a mouth)	Never
Sometimes (few times a mouth)	Never
Often (few times a week)	Never
Sometimes (few times a mouth)	Never
Sometimes (few times a mouth)	Never
Sometimes (few times a mouth)	Never
Sometimes (few times a mouth)	Never
Sometimes (few times a mouth)	Sometimes
Sometimes (few times a mouth)	Never
Sometimes (few times a mouth)	Never
Often (few times a week)	Never
Often (few times a week)	Never
Often (few times a week)	Sometimes
Often (few times a week)	Never
Sometimes (few times a mouth)	Never
Sometimes (few times a mouth)	Never
Sometimes (few times a mouth)	Never
Sometimes (few times a mouth)	Never
Sometimes (few times a mouth)	Never
Often (few times a week)	Never
Sometimes (few times a mouth)	Never
Often (few times a week)	Never
Sometimes (few times a mouth)	Never
Sometimes (few times a mouth)	Never
Regularly (few times a day)	Never
Often (few times a week)	Never
Sometimes (few times a mouth)	Never
Sometimes (few times a mouth)	Never
Often (few times a week)	Never
Often (few times a week)	Never
Often (few times a week)	Never
Often (few times a week)	Never
Often (few times a week)	Never
Often (few times a week)	Never
Often (few times a week)	Never
Sometimes (few times a mouth)	Sometimes
Often (few times a week)	Sometimes
Often (few times a week)	Never
Sometimes (few times a mouth)	Never
Often (few times a week)	Never
Often (few times a week)	Never
Often (few times a week)	Never
Often (few times a week)	Never
Often (few times a week)	Sometimes
Often (few times a week)	Never

Often (few times a week)	Never
Often (few times a week)	Never
Sometimes (few times a mouth)	Never
Often (few times a week)	Never
Often (few times a week)	Never
Often (few times a week)	Never
Often (few times a week)	Never
Often (few times a week)	Never
Often (few times a week)	Sometimes
Sometimes (few times a mouth)	Never
Sometimes (few times a mouth)	Never
Sometimes (few times a mouth)	Never
Sometimes (few times a mouth)	Never
Sometimes (few times a mouth)	Never
Sometimes (few times a mouth)	Never
Sometimes (few times a mouth)	Never
Often (few times a week)	Never
Often (few times a week)	Never
Often (few times a week)	Never
Sometimes (few times a mouth)	Never
Sometimes (few times a mouth)	Never
Often (few times a week)	Never
Often (few times a week)	Never
Sometimes (few times a mouth)	Sometimes
Sometimes (few times a mouth)	Never
Often (few times a week)	Never
Often (few times a week)	Never
Often (few times a week)	Never
Often (few times a week)	Never
Sometimes (few times a mouth)	Never
Sometimes (few times a mouth)	Never
Often (few times a week)	Never
Often (few times a week)	Never
Often (few times a week)	Never
Often (few times a week)	Sometimes
Often (few times a week)	Never
Often (few times a week)	Sometimes
Sometimes (few times a mouth)	Never
Sometimes (few times a mouth)	Never
Often (few times a week)	Never
Sometimes (few times a mouth)	Never
Often (few times a week)	Never
Sometimes (few times a mouth)	Never
Often (few times a week)	Never
Sometimes (few times a mouth)	Never
Often (few times a week)	Sometimes
Often (few times a week)	Sometimes
Sometimes (few times a mouth)	Never
Sometimes (few times a mouth)	Never
Sometimes (few times a mouth)	Never

Sometimes (few times a mouth)	Never
Often (few times a week)	Sometimes
Often (few times a week)	Never
Sometimes (few times a mouth)	Never
Sometimes (few times a mouth)	Sometimes
Sometimes (few times a mouth)	Never
Often (few times a week)	Sometimes
Sometimes (few times a mouth)	Never
Sometimes (few times a mouth)	Never
Often (few times a week)	Sometimes
Sometimes (few times a mouth)	Never
Sometimes (few times a mouth)	Never
Sometimes (few times a mouth)	Never
Sometimes (few times a mouth)	Never
Often (few times a week)	Never
Often (few times a week)	Sometimes
Sometimes (few times a mouth)	Never
Often (few times a week)	Never
Sometimes (few times a mouth)	Never
Sometimes (few times a mouth)	Never
Sometimes (few times a mouth)	Never
Sometimes (few times a mouth)	Never
Sometimes (few times a mouth)	Never
Often (few times a week)	Sometimes
Sometimes (few times a mouth)	Never
Often (few times a week)	Never
Sometimes (few times a mouth)	Never
Sometimes (few times a mouth)	Never
Often (few times a week)	Sometimes
Sometimes (few times a mouth)	Never
Sometimes (few times a mouth)	Never
Often (few times a week)	Never
Sometimes (few times a mouth)	Never
Often (few times a week)	Never
Often (few times a week)	Sometimes
Regularly (few times a day)	Never
Sometimes (few times a mouth)	Never
Sometimes (few times a mouth)	Never
Sometimes (few times a mouth)	Never
Sometimes (few times a mouth)	Never
Often (few times a week)	Sometimes
Often (few times a week)	Sometimes
Sometimes (few times a mouth)	Never
Often (few times a week)	Never
Sometimes (few times a mouth)	Never
Sometimes (few times a mouth)	Never
Sometimes (few times a mouth)	Sometimes
Often (few times a week)	Never
Sometimes (few times a mouth)	Regularly
Sometimes (few times a mouth)	Never

Often (few times a week)	Sometimes
Sometimes (few times a mouth)	Sometimes
Sometimes (few times a mouth)	Sometimes
Sometimes (few times a mouth)	Never
Sometimes (few times a mouth)	Never

21. Smoking status	22. Turn signal neglect?	23. Encroach car lanes?	24. Exceed speed limit?
Sometimes	Yes	Yes	Yes
Sometimes	No	No	No
Never	No	No	No
Regularly	No	No	No
Never	No	No	No
Never	No	No	No
Never	No	No	No
Sometimes	No	No	No
Never	No	No	No
Never	No	No	No
Never	No	No	No
Never	No	No	No
Sometimes	No	No	Yes
Sometimes	No	No	No
Never	No	No	No
Regularly	No	No	No
Sometimes	No	No	No
Never	No	Yes	Yes
Sometimes	No	No	No
Sometimes	No	No	No
Sometimes	No	No	No
Never	No	No	No
Sometimes	No	No	No
Sometimes	No	No	No
Never	No	No	No
Never	No	No	No
Sometimes	No	No	No
Never	No	No	No
Never	No	No	No
Never	No	No	No
Never	Yes	No	Yes
Regularly	No	No	No
Never	No	No	No
Never	No	No	No
Never	No	No	No
Regularly	No	No	No
Regularly	No	No	No
Never	No	No	No
Never	No	No	No
Sometimes	No	No	No
Never	No	No	Yes
Never	No	No	No
Sometimes	No	No	No
Never	No	No	No
Never	Yes	No	No
Regularly	Yes	No	Yes

Never	No	No	No
Never	No	No	No
Never	No	No	No
Never	No	No	No
Never	No	No	No
Regularly	No	No	No
Never	No	No	No
Regularly	No	No	No
Sometimes	No	No	No
Sometimes	No	No	No
Sometimes	No	No	No
Regularly	No	No	No
Never	No	No	No
Sometimes	No	No	No
Regularly	No	No	No
Regularly	No	No	No
Never	No	No	No
Sometimes	No	No	No
Regularly	No	No	No
Regularly	No	No	No
Never	No	No	No
Regularly	No	No	No
Never	No	No	No
Never	No	No	No
Never	No	No	No
Never	No	No	No
Sometimes	No	No	No
Regularly	No	No	No
Regularly	No	No	No
Never	No	No	Yes
Never	No	No	No
Never	No	No	No
Never	No	No	No
Regularly	No	No	No
Never	No	No	No
Never	No	No	No
Never	No	No	No
Never	No	No	No
Regularly	No	No	No
Never	No	No	No
Never	No	No	No
Never	No	No	No
Sometimes	No	No	Yes
Never	Yes	Yes	No
Never	No	No	No
Regularly	No	No	No
Regularly	No	No	No
Sometimes	No	No	No
Never	No	No	No
Never	No	No	No

Never	No	No	No
Never	No	No	No
Regularly	No	No	No
Sometimes	No	No	No
Never	No	No	No
Never	No	No	No
Never	No	No	No
Regularly	Yes	No	Yes
Regularly	No	No	No
Never	No	No	No
Never	No	No	No
Never	No	No	No
Never	Yes	No	No
Regularly	No	No	No
Never	No	No	No
Never	No	No	No
Never	No	No	No
Never	No	No	No
Regularly	No	No	No
Never	No	No	No
Regularly	No	No	No
Sometimes	Yes	No	No
Never	No	No	No
Never	No	No	No
Never	No	No	No
Never	No	No	No
Never	No	No	No
Sometimes	No	No	No
Regularly	No	No	No
Never	No	No	No
Regularly	Yes	No	Yes
Regularly	Yes	No	Yes
Never	No	No	No
Never	No	No	No
Never	No	No	No
Regularly	No	No	No
Regularly	No	No	No
Never	No	No	No
Regularly	No	No	No
Never	No	No	No
Regularly	No	No	No
Regularly	No	No	No
Never	No	No	No
Regularly	No	No	No
Never	No	No	No
Regularly	No	No	No
Never	No	No	No
Never	No	No	No
Never	No	No	No
Never	Yes	No	No

Never	No	No	No
Never	No	No	No
Never	Yes	No	No
Never	Yes	No	Yes
Never	Yes	No	No
Never	No	No	No
Never	No	No	No
Never	Yes	No	No
Never	Yes	No	No
Never	Yes	No	No
Sometimes	Yes	No	No
Never	No	No	No
Regularly	No	No	No
Regularly	No	No	No
Regularly	No	No	No
Never	No	No	No
Regularly	No	No	No
Never	No	No	No
Regularly	No	No	No
Sometimes	No	No	No
Never	No	No	No
Regularly	No	No	No
Regularly	No	No	No
Regularly	No	No	No
Never	No	No	No
Regularly	No	No	No
Sometimes	No	No	No
Regularly	No	No	No
Regularly	No	No	No
Sometimes	No	No	No
Regularly	No	No	No
Never	No	No	No
Never	Yes	No	No
Never	No	No	No
Never	No	No	No
Never	No	No	No
Never	No	No	No
Never	No	No	No
Regularly	No	No	No
Never	No	No	No
Regularly	No	No	No
Never	No	No	No
Regularly	No	No	No
Never	No	No	No
Never	No	No	No
Never	No	No	No
Regularly	No	No	No
Never	No	No	No
Regularly	Yes	Yes	Yes
Never	No	No	No

Regularly	No	No	No
Never	No	No	No
Never	No	No	No
Sometimes	No	No	No
Regularly	No	No	No
Never	No	No	No
Regularly	No	No	No
Never	No	No	No
Never	No	No	No
Never	No	No	No
Never	No	No	No
Never	No	No	Yes
Never	No	No	Yes
Never	No	No	Yes
Sometimes	No	No	No
Regularly	No	No	No
Never	No	No	No
Sometimes	No	No	No
Never	No	No	No
Regularly	No	No	No
Never	No	No	No
Regularly	No	No	No
Never	No	No	No
Never	No	No	Yes
Never	No	No	No
Never	No	No	No
Never	No	No	Yes
Sometimes	No	No	Yes
Never	No	No	Yes
Never	No	No	Yes
Sometimes	No	No	Yes
Regularly	No	No	Yes
Regularly	No	No	Yes
Never	No	No	Yes
Never	No	No	Yes
Never	No	No	Yes
Never	No	No	No
Regularly	No	No	No
Sometimes	No	No	No
Never	No	No	Yes
Never	No	No	Yes
Never	No	No	Yes
Never	No	No	Yes
Never	No	No	No
Regularly	No	No	No
Never	No	No	Yes
Regularly	No	No	Yes
Regularly	No	No	No
Regularly	No	No	Yes
Regularly	No	No	No

Never	No	No	No
Never	No	No	Yes
Never	No	No	No
Never	No	No	No
Never	No	No	No
Never	No	No	Yes
Never	No	No	No
Regularly	No	No	Yes
Never	No	No	No
Never	No	No	No
Regularly	No	No	Yes
Never	No	No	No
Regularly	No	No	No
Never	No	No	No
Never	No	No	No
Regularly	No	No	Yes
Regularly	No	No	No
Regularly	No	No	No
Never	No	No	Yes
Never	No	No	Yes
Never	No	No	No
Never	No	No	Yes
Never	No	No	Yes
Never	No	No	No
Never	No	No	Yes
Never	Yes	No	No
Never	No	No	Yes
Regularly	No	No	No
Never	No	No	Yes
Never	No	No	Yes
Regularly	No	No	No
Never	No	No	No
Never	No	No	Yes
Regularly	No	No	Yes
Regularly	No	No	No
Regularly	No	No	Yes
Never	No	No	Yes
Sometimes	No	No	Yes
Regularly	No	No	Yes
Regularly	No	No	Yes
Never	No	No	Yes
Never	No	No	Yes
Never	No	No	Yes
Never	No	No	Yes
Regularly	No	No	Yes
Never	No	No	Yes
Regularly	No	No	Yes
Regularly	No	No	Yes
Never	No	No	Yes
Regularly	No	No	No

Regularly	No	No	No
Regularly	Yes	No	Yes
Never	No	No	Yes
Never	No	No	No
Never	No	No	Yes
Regularly	No	No	No
Regularly	No	No	No
Regularly	No	No	No
Regularly	No	No	No
Regularly	No	No	No
Never	No	No	Yes
Never	No	No	No
Never	No	No	Yes
Regularly	No	No	Yes
Never	No	No	Yes
Never	No	No	No
Regularly	No	No	No
Regularly	No	No	No
Regularly	No	No	Yes
Regularly	No	No	Yes
Regularly	No	No	No
Regularly	No	No	No
Never	No	No	No
Never	No	No	No
Never	No	No	Yes
Never	No	No	No
Regularly	No	No	Yes
Never	No	No	No
Regularly	No	No	No
Never	No	No	No
Never	No	No	Yes
Never	No	No	No
Never	No	No	No
Sometimes	No	No	Yes
Sometimes	No	No	No
Regularly	No	No	Yes
Never	No	No	No
Never	No	No	Yes
Regularly	No	No	No
Regularly	No	No	No
Never	No	No	Yes
Never	No	No	Yes
Never	No	No	No
Regularly	No	No	Yes
Never	No	No	Yes
Sometimes	No	No	Yes
Never	No	No	Yes
Never	No	No	No
Never	No	No	No
Never	No	No	Yes

Never	No	No	Yes
Sometimes	No	No	No
Never	No	No	Yes
Never	No	No	Yes
Sometimes	No	No	No
Sometimes	No	No	Yes
Never	No	No	Yes
Never	No	No	Yes
Never	No	No	Yes
Never	No	No	No
Never	No	No	Yes
Never	No	No	Yes
Regularly	No	No	No
Never	No	No	Yes
Never	No	No	Yes
Never	No	No	No
Never	No	No	No
Never	No	No	Yes
Never	No	No	No
Never	No	No	Yes
Never	Yes	No	Yes
Never	No	No	Yes
Never	Yes	No	Yes
Never	No	No	Yes
Never	Yes	No	Yes
Never	No	No	No
Never	Yes	No	Yes
Never	No	No	No
Sometimes	No	No	Yes
Regularly	No	No	No
Never	No	No	No
Regularly	No	No	Yes
Never	No	No	No
Sometimes	No	No	No
Regularly	No	No	No
Sometimes	No	No	No
Never	No	No	Yes
Never	No	No	No
Never	No	No	Yes
Never	No	No	Yes
Regularly	No	No	No
Sometimes	No	No	No
Never	No	No	No
Regularly	No	No	Yes
Never	No	No	Yes
Never	No	No	No
Regularly	No	No	No
Never	No	No	No
Regularly	No	No	Yes
Never	No	No	No

Regularly	No	No	No
Regularly	No	No	No
Regularly	No	No	No
Never	No	No	No
Never	No	No	No

25. Red-light running?	26. Carry more than one passenger?	27. Smoke while driving?
Yes	Yes	Yes
Yes	No	No
Yes	Yes	No
Yes	No	No
Yes	No	No
Yes	No	No
Yes	No	No
Yes	No	No
Yes	No	No
Yes	Yes	No
Yes	No	No
Yes	No	No
No	Yes	Yes
Yes	No	No
Yes	No	No
Yes	No	No
Yes	No	No
Yes	Yes	No
Yes	Yes	No
Yes	Yes	No
Yes	No	No
Yes	No	No
Yes	No	No
Yes	No	No
Yes	No	No
Yes	No	No
Yes	No	No
Yes	No	No
Yes	Yes	No
Yes	Yes	No
Yes	Yes	Yes
Yes	No	Yes
No	No	No
Yes	No	No
No	No	No
Yes	No	No
Yes	No	No
No	No	No
Yes	No	No
Yes	No	No
Yes	Yes	No
Yes	Yes	No
Yes	Yes	Yes
No	Yes	No
Yes	No	No
Yes	Yes	Yes

Yes	No	No
Yes	No	No
Yes	No	No
Yes	No	No
Yes	No	No
Yes	No	No
Yes	Yes	No
Yes	Yes	No
Yes	Yes	No
Yes	No	No
Yes	Yes	No
Yes	No	No
Yes	No	No
Yes	No	Yes
Yes	Yes	No
Yes	No	No
Yes	No	No
Yes	No	Yes
Yes	No	Yes
Yes	No	No
Yes	Yes	No
Yes	Yes	No
Yes	Yes	Yes
Yes	Yes	No
Yes	No	No
Yes	No	No
Yes	No	No
Yes	Yes	No
Yes	Yes	No
No	Yes	No
Yes	No	No
Yes	Yes	No
Yes	No	No
Yes	No	No
Yes	No	No
Yes	No	No
Yes	Yes	No
Yes	Yes	No
Yes	No	No
Yes	No	No
Yes	Yes	No
Yes	No	No
Yes	Yes	Yes
Yes	No	No
Yes	Yes	No
Yes	Yes	No
Yes	Yes	No
Yes	No	No
Yes	No	No
Yes	Yes	No

Yes	No	No
Yes	Yes	No
Yes	No	No
Yes	No	No
Yes	Yes	No
Yes	No	No
Yes	Yes	No
No	No	Yes
No	Yes	No
Yes	Yes	No
Yes	No	No
Yes	No	No
No	Yes	Yes
Yes	Yes	No
Yes	Yes	No
Yes	No	No
Yes	Yes	No
Yes	No	No
Yes	Yes	No
Yes	Yes	No
Yes	No	No
Yes	Yes	No
Yes	Yes	No
Yes	No	No
Yes	Yes	No
Yes	No	No
Yes	Yes	No
Yes	No	No
Yes	No	No
Yes	No	No
No	Yes	Yes
Yes	Yes	Yes
Yes	No	No
Yes	Yes	No
Yes	No	No
Yes	Yes	No
Yes	No	No
Yes	Yes	No
Yes	Yes	No
Yes	No	No
Yes	Yes	No
Yes	No	No
Yes	Yes	No
Yes	No	No
Yes	No	No
Yes	No	No
Yes	Yes	No
Yes	Yes	No
Yes	Yes	Yes
Yes	No	No

Yes	Yes	No
Yes	Yes	No
Yes	No	No
Yes	Yes	No
Yes	Yes	No
Yes	No	No
Yes	Yes	No
Yes	Yes	No
Yes	No	No
Yes	Yes	No
Yes	Yes	No
Yes	Yes	No
Yes	No	No
Yes	Yes	No
Yes	No	Yes
Yes	No	No
Yes	No	No
Yes	No	No
Yes	No	No
Yes	No	No
Yes	No	No
Yes	Yes	No
Yes	Yes	No
Yes	Yes	No
Yes	Yes	No
Yes	No	No
Yes	Yes	No
Yes	Yes	No
Yes	Yes	No
Yes	Yes	No
Yes	No	No
Yes	Yes	No
Yes	No	No
No	No	No
Yes	No	No
Yes	Yes	No
Yes	No	No
Yes	No	No
Yes	No	No
Yes	No	No
Yes	No	No
Yes	No	No
Yes	Yes	No
Yes	No	No
Yes	No	No
Yes	No	No
Yes	Yes	No
Yes	No	No
Yes	No	No
Yes	No	No

Yes	No	No
No	No	No
No	No	No
No	No	No
Yes	No	Yes
Yes	No	No
Yes	Yes	No
Yes	No	No
Yes	No	No
Yes	No	No
Yes	Yes	No
Yes	No	
Yes	No	Yes
Yes	No	Yes
Yes	No	Yes
Yes	No	Yes
Yes	No	Yes
Yes	No	No
Yes	No	Yes
Yes	No	Yes
Yes	Yes	Yes
Yes	No	Yes
Yes	No	Yes
Yes	No	Yes
Yes	No	Yes
Yes	No	Yes
Yes	Yes	Yes
Yes	No	Yes
Yes	No	Yes
Yes	Yes	Yes
Yes	No	Yes
Yes	No	Yes
Yes	No	Yes
Yes	No	Yes
Yes	No	No
Yes	No	No
Yes	Yes	Yes
Yes	Yes	Yes
Yes	No	No
Yes	No	No
Yes	No	No
Yes	No	Yes
Yes	No	Yes
Yes	No	No
Yes	No	Yes
Yes	Yes	Yes
Yes	Yes	Yes
Yes	No	Yes
Yes	No	No
Yes	No	Yes

Yes	No	Yes
Yes	No	No
Yes	No	No
Yes	No	Yes
Yes	No	No
Yes	No	No
Yes	No	No
Yes	Yes	Yes
Yes	No	Yes
Yes	No	Yes
Yes	No	No
Yes	No	No
Yes	No	No
Yes	No	Yes
Yes	No	No
Yes	Yes	Yes
Yes	No	Yes
Yes	No	Yes
Yes	Yes	Yes
Yes	No	Yes
Yes	Yes	Yes
Yes	No	Yes
Yes	No	Yes
Yes	No	Yes
Yes	No	Yes
Yes	No	Yes
Yes	No	Yes
Yes	No	Yes
Yes	Yes	Yes
Yes	Yes	Yes
Yes	No	Yes
Yes	Yes	Yes
Yes	No	Yes
Yes	No	No
Yes	No	Yes
Yes	Yes	Yes
Yes	No	Yes
Yes	No	Yes
Yes	No	Yes
Yes	Yes	Yes
Yes	No	Yes
Yes	No	Yes
Yes	No	Yes
Yes	No	Yes
Yes	No	Yes
Yes	No	No
Yes	Yes	Yes
Yes	No	No
Yes	No	No
Yes	No	Yes

Yes	No	Yes
Yes	No	Yes
Yes	No	No
Yes	No	Yes
Yes	No	Yes
Yes	No	Yes
Yes	No	Yes
Yes	No	Yes
Yes	No	No
Yes	Yes	Yes
Yes	Yes	Yes
Yes	No	Yes
Yes	No	Yes
Yes	Yes	Yes
Yes	Yes	No
Yes	Yes	Yes
Yes	No	Yes
Yes	No	Yes
Yes	Yes	No
Yes	Yes	Yes
Yes	Yes	Yes
Yes	No	No
Yes	Yes	No
Yes	No	Yes
Yes	Yes	Yes
Yes	No	No
Yes	No	Yes
Yes	No	Yes
Yes	No	Yes
Yes	No	Yes
Yes	Yes	No
Yes	Yes	Yes
Yes	No	No
Yes	No	No
Yes	Yes	Yes
Yes	Yes	Yes
Yes	No	No
Yes	No	No
Yes	Yes	Yes
Yes	No	Yes
Yes	Yes	Yes
Yes	No	Yes
Yes	No	Yes
Yes	Yes	Yes
Yes	Yes	Yes
Yes	Yes	Yes
Yes	No	Yes
Yes	No	Yes
Yes	No	No
Yes	Yes	Yes

Yes	No	Yes
Yes	No	No
Yes	No	Yes
Yes	No	Yes
Yes	No	No
Yes	No	Yes
Yes	Yes	Yes
Yes	No	Yes
Yes	No	Yes
Yes	No	Yes
Yes	Yes	No
Yes	No	No
Yes	No	Yes
Yes	Yes	No
Yes	No	No
Yes	No	No
Yes	No	Yes
Yes	No	No
Yes	No	Yes
Yes	No	Yes
Yes	No	Yes
Yes	No	Yes
Yes	No	Yes
Yes	Yes	Yes
Yes	No	Yes
Yes	No	No
Yes	No	Yes
Yes	No	Yes
Yes	No	No
Yes	Yes	No
Yes	No	No
Yes	Yes	Yes
Yes	No	No
Yes	No	No
Yes	Yes	No
Yes	No	No
Yes	Yes	No
Yes	No	Yes
Yes	No	No
Yes	No	No
Yes	No	No
Yes	No	No
Yes	No	Yes
Yes	No	No
Yes	No	Yes
Yes	No	No
Yes	No	No
Yes	No	No
Yes	Yes	No
Yes	No	Yes

Yes	No	No
Yes	No	No
Yes	No	No
Yes	No	No
Yes	Yes	No

28. Use helmet?	29. Reckless overtaking?	30. Drink driving?
Yes	Yes	Yes
Yes	No	No
No	Yes	No
Yes	No	No
Yes	No	No
Yes	No	No
Yes	No	No
No	No	No
Yes	No	No
Yes	No	No
No	No	No
Yes	No	No
Yes	Yes	Yes
Yes	No	No
Yes	No	No
Yes	No	No
Yes	No	No
Yes	No	No
Yes	No	No
Yes	No	No
Yes	No	No
Yes	No	No
Yes	No	No
Yes	No	No
Yes	No	No
Yes	No	No
Yes	No	No
Yes	No	No
Yes	No	No
No	No	No
Yes	No	Yes
Yes	No	No
Yes	No	No
No	No	No
Yes	No	No
Yes	No	No
No	No	No
Yes	No	No
Yes	No	No
Yes	No	No
Yes	Yes	No
Yes	No	No
Yes	No	Yes
Yes	No	No
Yes	No	No
Yes	Yes	Yes

Yes	No	No
Yes	No	No
Yes	No	No
Yes	No	No
Yes	No	No
No	No	No
Yes	No	No
Yes	No	No
Yes	No	No
No	No	No
Yes	No	No
Yes	No	No
Yes	No	No
No	No	No
Yes	No	No
Yes	No	No
Yes	No	No
No	No	No
No	No	Yes
Yes	No	No
No	No	No
No	No	No
Yes	No	No
No	No	No
No	No	No
Yes	No	No
Yes	No	No
Yes	No	No
Yes	No	No
Yes	No	No
Yes	No	No
No	No	No
Yes	No	No
Yes	No	No
No	No	No
Yes	No	No
Yes	No	No
Yes	No	No
Yes	No	No
Yes	No	No
No	No	No
Yes	No	No
Yes	No	Yes
Yes	No	No
Yes	No	No
No	No	No
Yes	No	No
Yes	No	No
Yes	No	No
Yes	No	No

Yes	No	No
No	No	No
Yes	No	No
No	No	No
Yes	No	No
Yes	No	No
Yes	No	No
Yes	Yes	Yes
Yes	No	No
Yes	No	No
Yes	No	No
No	No	No
Yes	No	No
Yes	No	No
Yes	No	No
No	No	No
No	No	No
Yes	No	No
Yes	No	No
Yes	No	No
Yes	No	No
Yes	No	No
No	No	No
Yes	No	No
No	No	No
Yes	No	No
Yes	No	No
Yes	No	No
Yes	No	No
Yes	No	
Yes	No	Yes
Yes	Yes	Yes
Yes	No	No
Yes	No	No
Yes	No	No
No	No	No
Yes	No	No
Yes	No	No
No	No	No
Yes	No	No
No	No	No
Yes	No	No
Yes	No	No
No	No	No
Yes	No	No
Yes	No	No
No	No	No
Yes	No	No
Yes	No	No
Yes	No	No

Yes	No	No
Yes	No	No
Yes	No	No
Yes	No	No
Yes	No	No
Yes	No	No
Yes	No	No
Yes	No	No
Yes	No	No
Yes	No	No
No	No	No
Yes	No	No
Yes	No	No
No	No	No
Yes	No	No
Yes	No	No
Yes	No	No
Yes	No	No
Yes	No	No
Yes	No	No
Yes	No	No
No	No	No
No	No	No
No	No	No
No	No	No
Yes	No	No
Yes	No	No
Yes	No	No
Yes	No	No
No	No	No
Yes	No	No
Yes	No	No
Yes	No	No
Yes	No	No
Yes	No	No
No	No	No
Yes	No	No
Yes	No	No
No	No	No
Yes	No	No
Yes	No	No
Yes	No	No
No	No	No
Yes	No	No
Yes	No	No
Yes	No	No
No	No	No
Yes	No	No
Yes	No	No
Yes	No	No

Yes	No	No
Yes	No	No
Yes	No	No
Yes	No	No
Yes	No	No
Yes	No	No
No	No	No
Yes	No	No
Yes	No	No
Yes	No	No
Yes	No	No
No	No	No
No	No	No
Yes	No	No
No	No	No
Yes	No	No
No	No	No
No	No	No
No	No	No
Yes	No	No
No	No	No
No	No	No
No	No	No
No	No	No
No	No	No
No	No	No
No	No	No
No	No	No
Yes	No	No
No	No	No
No	No	No
Yes	No	No
Yes	No	No
No	No	No
No	No	No
No	No	No
No	No	No
No	No	No
No	No	No
Yes	No	No
No	No	No
Yes	No	No
No	No	No
No	No	No
No	No	No
No	No	No
No	No	No
No	No	No
No	No	No
No	No	No

Yes	No	No
No	No	No
No	No	No
No	No	No
No	No	No
No	No	No
No	No	No
No	No	No
No	No	No
No	No	No
No	No	No
No	No	No
No	No	No
Yes	No	No
No	No	No
No	No	No
No	No	No
No	No	No
No	No	No
No	No	Yes
No	No	No
No	No	No
No	Yes	No
No	No	No
No	No	No
No	No	No
Yes	No	No
No	No	No
No	No	No
No	No	No
No	No	No
No	No	No
No	No	No
No	No	No
No	No	No
No	No	No
No	No	No
No	No	No
No	No	No
No	No	No
No	No	No
Yes	No	No
Yes	No	No
No	No	No
No	No	No
No	No	No
Yes	No	No
No	No	No
No	No	No
No	No	No

No	No	No
No	No	No
No	No	No
No	No	No
No	No	No
No	No	No
No	No	No
Yes	No	No
No	No	No
No	No	No
Yes	No	No
No	No	No
Yes	No	No
Yes	No	No
No	No	No
No	No	No
Yes	No	No
No	No	No
No	No	No
No	No	No
No	No	No
No	No	No
No	No	No
No	No	No
Yes	No	No
No	No	No
No	No	No
No	No	No
Yes	No	No
Yes	No	No
No	No	No
No	No	No
No	No	No
No	No	No
No	No	No
Yes	No	No
No	No	No
No	No	No
No	No	No
No	No	No
No	No	No
Yes	No	No
No	No	No
No	No	No
No	No	No
Yes	No	No
Yes	No	No
Yes	No	No
No	No	No
Yes	No	No

Yes	No	No
No	No	No
Yes	No	No
Yes	No	No
No	No	No
No	No	No
No	No	No
No	No	No
Yes	No	No
No	No	No
No	No	No
No	No	No
No	No	No
No	No	No
No	No	No
Yes	No	No
No	No	No
No	No	No
No	No	No
Yes	No	No
No	No	No
No	No	No
No	No	No
Yes	No	No
No	No	No
No	No	No
No	No	No
No	No	No
No	No	No
No	No	No
No	No	No
Yes	No	No
No	No	No
No	No	No
Yes	No	No
No	No	No
No	No	No
No	No	No
No	No	No
No	No	No
No	No	No
No	No	No
No	No	No
Yes	No	No
Yes	No	No
No	No	No
Yes	No	No
No	No	No
No	No	No
No	No	No

No	No	No
No	No	No
No	No	No
No	No	No
No	No	No
